# MRI‐based strain measurements reflect morphological changes following myocardial infarction: A study on the UK Biobank cohort

**DOI:** 10.1111/joa.13787

**Published:** 2022-12-09

**Authors:** Doyin S. Mansell, Eva Sammut, Vito D. Bruno, Raimondo Ascione, Jonathan C. L. Rodrigues, Harinderjit S. Gill, Katharine H. Fraser, Andrew N. Cookson

**Affiliations:** ^1^ Department of Mechanical Engineering University of Bath Bath UK; ^2^ Department of Translational Science, Bristol Heart Institute and Translational Biomedical Research Centre, Faculty of Health Science, Bristol Royal Infirmary, Level 7 University of Bristol Bristol UK; ^3^ Department of Radiology Royal United Hospitals Bath NHS Foundation Trust Bath UK; ^4^ Department of Health University of Bath Bath UK; ^5^ Centre for Therapeutic Innovation University of Bath Bath UK

## Abstract

In a porcine experimental model of myocardial infarction, a localised, layer‐specific, circumferential left ventricular strain metric has been shown to indicate chronic changes in ventricular function post‐infarction more strongly than ejection fraction. This novel strain metric might therefore provide useful prognostic information clinically. In this study, existing clinical volume indices, global strains, and the novel, layer‐specific strain were calculated for a large human cohort to assess variations in ventricular function and morphology with age, sex, and health status. Imaging and health data from the UK Biobank were obtained, including healthy volunteers and those with a history of cardiovascular illness. In total, 710 individuals were analysed and stratified by age, sex and health. Significant differences in all strain metrics were found between healthy and unhealthy populations, as well as between males and females. Significant differences in basal circumferential strain and global circumferential strain were found between healthy males and females, with males having smaller absolute values for both (all p≤ 0.001). There were significant differences in the functional variables left ventricular ejection fraction, end‐systolic volume, end‐systolic volume index and mid‐ventricular circumferential strain between healthy and unhealthy male cohorts aged 65–74 (all p≤ 0.001). These results suggest that whilst regional circumferential strains may be useful clinically for assessing cardiovascular health, care must be taken to ensure critical values are indexed correctly to age and sex, due to the differences in these values observed here.

## Graphical abstract

Boxplot showing statistically significant differences in basal circumferential strains between healthy males and females, particularly for age ranges 45‐54 and 65‐74. Based on an analysis of 710 participants in the UK Biobank, the major findings of this study were that healthy males have lower basal and global circumferential left ventricular strains than healthy females. Comparing healthy to unhealthy participants also found increased mid‐ventricular circumferential strains, lowered ejection fractions, and increased end‐systolic volumes in the unhealthy group, particularly for males aged 65‐74.

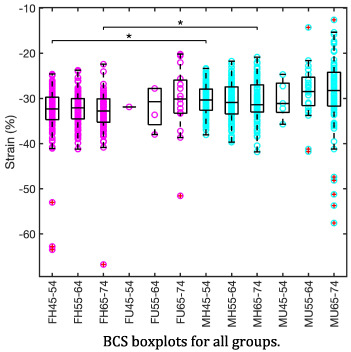

**Abbreviations**
ACS: apical circumferential strain
ANOVA: analysis of variance
BCS: basal circumferential strain
BMI: body mass index
BSA: body surface area
CMR: cardiovascular magnetic resonance
ED: end diastole
EDV: end diastolic volume
ESV: end systolic volume
ESVi: end systolic volume index
GCS: global circumferential strain
HF: heart failure
HR: heart rate
LV: left ventricular
LVEF: left ventricular ejection fraction
MCS: mid‐circumferential strain
MI: myocardial infarction
MRI: magnetic resonance imaging
NSTEMI: non‐ST‐elevation myocardial infarction
STE: speckle tracking echocardiography
STEMI: ST‐elevation myocardial infarction

## 1 INTRODUCTION

Left ventricular (LV) strain is a useful tool for the prediction of outcomes following myocardial infarction (MI) and other cardiovascular illnesses (Gavara et al., 2017; Koos et al., 2013; Moen et al., 2011; Rademakers & Nagel, 2018). It can provide supplementary information about changes to LV function alongside other standard clinical indices derived from medical imaging. These indices may provide morphological information such as changes to end‐diastolic volume (EDV) and end‐systolic volume (ESV) and functional information such as stroke volume and left ventricular ejection fraction (LVEF). Other factors used in such an assessment of MI include infarct size, age, sex and body size. There is a need to understand the links between age, sex or cardiovascular health status, and typical strain values for these populations, as well as how these strain values relate to indices currently used clinically. This would provide scientific insights into differences in cardiac function between sub‐groups and provide a firm basis for the use of strains in clinical practice.

Cardiovascular magnetic resonance imaging (CMR) is the gold standard for imaging of cardiovascular morphology and function but is expensive and time‐consuming when compared with echocardiography, which is fast, inexpensive and ubiquitous in healthcare clinics. For these reasons, large‐scale studies on cardiovascular function are often performed using echocardiography. Due to difficulties associated with speckle tracking echocardiography (STE), such as poor spatial resolution, and operator‐induced errors in transducer positioning, it is preferable to use CMR. Large‐scale studies on strain have been conducted (Stpylen et al., 2019), but fewer have used CMR, with studies often having an upper limit of 150–200 participants (Andre et al., 2015; Augustine et al., 2013; Koos et al., 2013; Mangion et al., 2016; Rodriguez‐Palomares et al., 2019). Many studies also focus primarily on the longitudinal strain, as there is evidence it is a strong prognosticator (Gavara et al., 2017). Indeed, it has often been found to be a stronger prognosticator than circumferential or radial strains, with Rademakers and Nagel explaining that whilst some have argued that this is because it is more affected by the damage caused by MI as longitudinal fibres are more prevalent at the endocardium (Streeter et al., 1969), in their view this is unlikely as the majority of fibres throughout the myocardium are obliquely oriented across the wall (ibid.), thus contributing to both longitudinal and circumferential strains (Rademakers & Nagel, 2018). An alternative explanation is that longitudinal strain is no more representative than short‐axis strain but that poor imaging and contouring in the short‐axis can have a larger impact on reproducibility and outcome (ibid.). Through‐plane motion is another factor that may affect short‐axis strains more than long‐axis‐based measures (Claus et al., 2015).

The particular strain calculation approach used also affects this; imaging methods such as feature tracking, tagged MRI, strain‐encoded CMR, speckle tracking are used and then whether myocardial strain or layer‐specific strains are calculated is also of concern. Due to these differences in results caused by differing imaging modalities, imaging machines and even research centre practices, no overarching consensus values or reference ranges for strains have been reached. It has been noted that relationships between strain and other factors such as age or body size would more likely be universal across these imaging modalities (Støylen et al., 2019). Furthermore, there are few large‐scale studies which include both healthy and unhealthy populations, with many studies opting for either only healthy populations, or studying only a specific illness.

In previous works (Mansell et al., 2020; Mansell et al., 2021) a localised, layer‐specific, circumferential LV strain metric was developed, using porcine imaging data. The reproducibility of this strain metric was demonstrated across different users and software packages and compared favourably to that of standard clinical metrics such as global strains and ejection fraction (Mansell et al., 2020). Its ability to characterise acute changes to LV function post MI was similar to volume metrics such as ejection fraction. However, in that study it was found that these changes in strain also persisted to the chronic period more strongly than changes in ejection fraction, suggesting that strains may provide additional information on ventricular remodelling and function (Mansell et al., 2021).

The aim of this study was to determine if there are differences in strain, as quantified by the novel layer‐specific circumferential strain metric, between different population subgroups stratified by age, sex and cardiovascular health status, and assess how these functional differences compare to morphological differences between groups. To achieve this aim, strains and other clinical metrics were calculated for a large population of volunteers within the UK Biobank.

## 2 METHODS

### 2.1 Data source

Cardiovascular MRI and relevant patient history data were obtained from the UK Biobank, a large‐scale biomedical database containing health information for over half a million UK participants (https://www.ukbiobank.ac.uk). Permission was obtained to access data for use in this project (application number 52530, Odunmbaku‐Mansell, 2020). Ethical implications associated with this study were considered in line with university regulations (EIRA1 number 2920). Further information about UK Biobank ethics can be found online (Biobank, 2007).

### 2.2 CMR protocol

For cardiac function, the UK Biobank's CMR acquisitions included three long‐axis cines (2‐, 3‐ and 4‐chamber orientations), and a complete short‐axis stack covering the left (and right) ventricle (Petersen et al., 2016). Imaging was performed in Cheadle, United Kingdom, using a 1.5 T scanner (MAGNETOM Aera, Syngo Platform VD13A, Siemens Healthcare). The acquisition parameters of interest to this study were as follows: slice thickness of 8 mm, slice gap of 2 mm and temporal resolution of 31.56 ms.

### 2.3 Image analysis and segmentation

CVI42 (Release 5.11.2, Circle Cardiovascular Imaging Inc.) was used to extract endocardial contours automatically using the in‐built machine learning‐based segmentation algorithm. Contour lengths and enclosed areas were extracted from the segmentation at the locations corresponding to imaging slice positions. All contours were checked by an experienced reader, with only 636 of the 29,826 segmented contours (2.13%) needing manual correction.

### 2.4 Deformation analysis

The novel method for calculating strains proposed by Mansell et al. (Mansell et al., 2020; Mansell et al., 2021) was used here. Endocardial circumferential strains, $\varepsilon_{n}$, were calculated from the perimeter length data extracted from the segmentations produced by CVI42, using the definition in Equation 1:

(1) $$\varepsilon_{n}=\frac{L_{n}-L_{0}}{L_{0}}$$

where $L_{0}$ is the endocardial perimeter reference length at end diastole (ED), and $L_{n}$ the endocardial perimeter length at time n throughout the cardiac cycle for a given cine slice. The short‐axis slices were divided into three ‘vertical’ regions in the LV: apex, mid‐ventricle and base. The number of slices in each region varied depending on the total number of slices for a given patient. The minimum total number of slices was 6 and the maximum 10. Correspondingly, the minimum number of slices in a region was 2 and the maximum 4. The mean of the circumferential strains in each region was then calculated. Global circumferential strain (GCS) was also calculated as the mean of the circumferential strains from all slices (Mansell et al., 2020).

Though volumetric measures could have also been calculated using the enclosed areas of the contours and known slice thickness, these values had been calculated and verified in other studies and supplied to the UK Biobank. As such, the values from the UK Biobank were used.

### 2.5 Data selection

#### 2.5.1 Study population

Participants in the UK Biobank database who had short‐axis CMR images were assessed. Participants with non‐white British or Irish backgrounds, diabetes, current or ex‐tobacco smokers, with a BMI outside of the range 18.5–30 $\mathrm{kg}/m^{2}$ and aged outside of the range 45–74 years old were excluded from the analysis. The comorbidities listed were similar (though less restrictive) to those from a study which investigated reference ranges in baseline cardiovascular health of the population (Petersen et al., 2017) and comorbidities such as hypertension, respiratory disease and renal disease. These exclusions were chosen mainly to allow a more direct comparison with the results of Petersen et al. Exclusions based on ethnicity were unfortunately necessary due to insufficient numbers of patients with non‐white backgrounds included in the Biobank dataset for statistically significant conclusions to be drawn. Altogether, these exclusions resulted in 12,398 potential participants being identified.

Participants were split into groups by sex, age (45–54, 55–64 or 65–74 years) and health status (those with known cardiovascular illnesses and those without) to give 12 groups in total. These age ranges were chosen to enable easy comparison with similar studies. Cardiovascular illnesses were defined as STEMI (ST‐elevation myocardial infarction), NSTEMI (non‐ST‐elevation myocardial infarction) and HF (heart failure) as documented in the UK Biobank data, though the degree to which HF had progressed was not specified in the dataset. Those without cardiovascular illnesses gave a revised total of 12,866 participants to choose from and were distributed by age ranges as shown in Table 1. As there was a sufficiently large pool of data, 100 participants were randomly selected from each of the three age groups for both males and females. The group of volunteers with known cardiovascular illnesses gave a total of 136 participants and were distributed by age range as shown in Table 1. Of those participants, 56 had suffered STEMI (50 males, 6 females), 64 had suffered from NSTEMI (50 males, 14 females) and 12 had suffered heart failure (8 males, 4 females).

**TABLE 1:** Number of healthy and unhealthy (British white and Irish) potential participants in the UK Biobank repository in each age range by sex, and numbers of participants selected for this study

Age range	Total *N*		*N* males		*N* females	
	*N* Biobank	*N* study	*N* Biobank	*N* study	*N* Biobank	*N* study
Healthy cohort						
45–54	1988	194	828	100	1160	94
55–64	5133	190	2099	96	3034	94
65–74	5191	190	2324	97	2867	93
Unhealthy cohort						
45–54	7	6	5	5	2	1
55–64	31	28	25	24	6	4
65–74	98	97	82	82	16	15

### 2.6 Statistical analysis

Statistical analyses were run on all variables of interest: apical circumferential strain (ACS), mid‐circumferential strain (MCS), basal circumferential strain (BCS), GCS, LVEF, EDV, ESV, end‐systolic volume index (ESVi) and body surface area (BSA).

To assess the hypothesis that there is a change in functional metrics (strain and volumetric measures) between healthy and unhealthy groups, as well as the hypothesis that there is a difference in the metrics between men and women, statistical analyses were performed. An initial set of Student's *t*‐tests were performed for all variables (ESV, EDV, LVEF, ESVi, BSA, ACS, MCS, BCS, GCS), and data were grouped simply: all healthy vs. all unhealthy volunteers (men and women), and all male vs. all female volunteers (irrespective of health status). p≤ 0.05 was considered to be statistically significant.

A further hypothesis was that there would be changes in the metrics between age groups within the sexes for both healthy and unhealthy populations. An initial one‐way ANOVA was conducted for each variable (ESV, EDV, LVEF, ESVi, BSA, ACS, MCS, BCS, GCS). If the results of the ANOVA indicated that there was some statistical significance to p= 0.05, further tests were conducted to see where the differences lay. Planned contrasts between selected subgroups were performed to avoid unnecessary comparisons being performed. These planned contrasts were Female Healthy 45–54 vs Female Healthy 55–64, Female Healthy 45–54 vs Female Healthy 65–74, Female Healthy 55–64 vs Female Healthy 65–74, Female Healthy 45–54 vs Male Healthy 45–54, Female Healthy 55–64 vs Male Healthy 55–64, Female Healthy 65–74 vs Male Healthy 65–74, Male Healthy 45–54 vs Male Healthy 55–64, Male Healthy 45–54 vs Male Healthy 65–74, Male Healthy 55–64 vs Male Healthy 65–74, Male Healthy 65–74 vs Male Unhealthy 65–74, Female Healthy 65–74 vs Female Unhealthy 65–74, Male Healthy 55–64 vs Male Unhealthy 55–64, Male Unhealthy 55–64 vs Male Unhealthy 65–74, All Males Healthy vs All Females Healthy, All Females Healthy vs All Females Unhealthy, All Males Healthy vs All Males Unhealthy and All Females Unhealthy vs. All Males Unhealthy. To account for any non‐orthogonal contrasts and in order to control the familywise Type I error rate, a Bonferroni correction was used meaning that p≤0.003 was defined as significant. Bootstrapping was employed during these analyses, as it is a robust method of estimating the properties of the sample distribution from the sample data.

Outliers were defined as being outside ±2.7 standard deviations from the mean. In two cases, it was evident that values for the data were unphysical in nature (LV volumes which were 8–10 times greater than expected), and had been inputted incorrectly by the UK Biobank. After removing five outlier individuals, means, medians, standard deviations and 95% confidence intervals were calculated for all groups. The final number of participants used in the study are presented in Table 1. Not all data from the UK Biobank came with all entries included, and as such, the values listed in this table are the maximum possible N for any group for any variable.

Pearson correlation coefficients between all variables of interest (EDV, ESV, ESVi, LVEF, ACS, MCS, BCS, GCS) and both age and BSA were calculated via linear regression. For these correlations, participants were ‘lumped’ into their larger groups, i.e. all healthy females, all healthy males, all unhealthy females and all unhealthy males. A p≤ 0.05 was considered to be statistically significant and bootstrapping was again used to analyse the results from the correlations. All statistical analyses were conducted using IBM SPSS (IBM SPSS Statistics for Windows, Version 25.0, IBM Corp.).

## 3 RESULTS

### 3.1 Population characteristics

Characteristics for all participants are provided in Table 2. More detailed baseline characteristics, which are stratified by gender and health status, are presented in Tables S5–S8. For the unhealthy groups, the mean time since the first cardiac event (any damage done to cardiac tissue) is shown in Table S9.

**TABLE 2:** Characteristics for all healthy and unhealthy participants stratified by age group

Characteristic	Age group (years)					
	45–54		55–64		65–74	
	Healthy	Unhealthy	Healthy	Unhealthy	Healthy	Unhealthy
*N*	194	6	190	28	190	97
Age (years)	52.0 (±2.0)	53.0 (±1.0)	60.0 (±3.0)	61.0 (±3.0)	69.0 (±3.0)	69.0 (±3.0)
Male Gender (*N*[%])	100 (51.5)	5 (83.3)	96 (50.5)	24 (71.4)	97 (51.1)	82 (69.1)
HR (bpm)	59.9 (±8.0)	53.6 (±11.2)	61.1 (±9.4)	57.0 (±10.4)	62.7 (±9.6)	57.0 (±9.2)
BMI ($\mathrm{kg}/m^{2}$)	25.4 (±2.6)	27.3 (±2.1)	24.8 (±2.7)	25.0 (±2.5)	25.1 (±2.6)	25.6 (±2.3)
Weight (kg)	74.9 (±12.1)	76.7 (±6.2)	72.4 (±11.9)	75.9 (±8.9)	72.1 (±10.7)	75.9 (±10.5)
ESV (ml)	58.7 (±15.1)	59.3 (±13.7)	54.6 (±14.9)	67.1 (±24.3)	52.1 (±14.5)	67.2 (±26.4)
EDV (ml)	151.6 (±31.1)	170.6 (±22.8)	141.2 (±30.9)	169.8 (±25.6)	136.3 (±30.9)	158.8 (±41.6)
LVEF (%)	56.2 (±5.0)	53.4 (±6.2)	56.8 (±5.3)	52.0 (±7.4)	55.9 (±5.8)	50.4 (±8.1)
ESVi ($\mathrm{ml}/m^{2}$)	35.4 (±7.5)	43.7 (±10.9)	33.1 (±7.3)	43.4 (±12.3)	33.0 (±8.1)	42.3 (±15.4)
BSA ($m^{2}$)	1.9 (±0.2)	1.8 (±0.1)	1.8 (±0.2)	1.9 (±0.1)	1.8 (±0.2)	1.9 (±0.2)
ACS (%)	−41.7 (±7.4)	−36.7 (±10.3	−41.9 (±8.0)	−40.2 (±14.6)	−41.6 (±8.1)	−37.8 (±13.8)
MCS (%)	−28.5 (±3.6)	−29.0 (±5.2)	−29.6 (±4.3)	−28.3 (±8.4)	−30.0 (±4.4)	−26.8 (±6.8)
BCS (%)	−31.6 (±5.0)	−30.5 (±3.9)	−31.5 (±3.9)	−29.2 (±5.8)	−31.8 (±4.8)	−29.3 (±8.0)
GCS (%)	−33.4 (±3.9)	−31.6 (±5.9	−33.7 (±4.4)	−31.6 (±8.2)	−33.8 (±4.6)	−30.6 (±7.7)

### 3.2 Statistical analysis

Results from the Student's *t*‐test were all statistically significant to *p* ≤ 0.05 (see Table S1), with all volume indices, BSA, BCS and GCS, particularly strongly significant. For auxiliary data from the *t*‐tests please see Tables S2 and S3.

**FIGURE 1:**
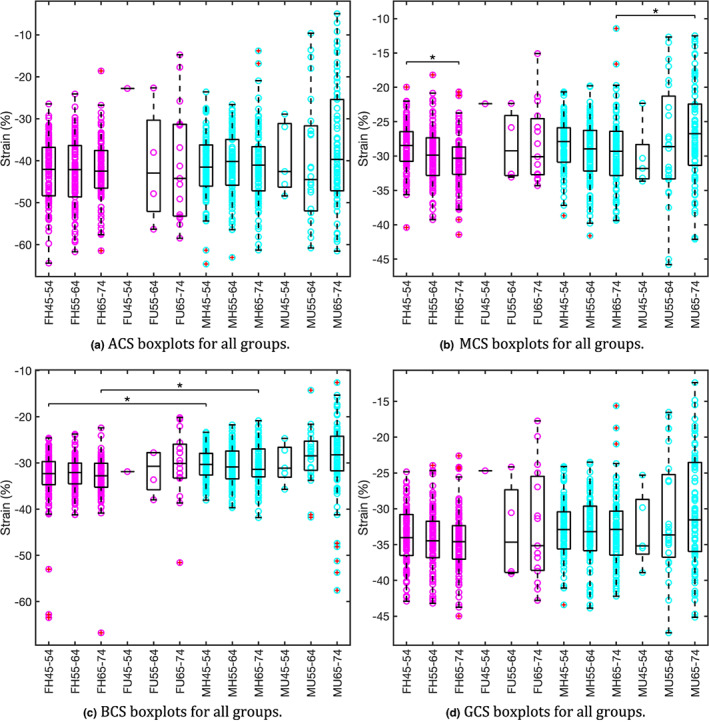
Strain boxplots for all groups: M = male; F = female; H = healthy; U = unhealthy; XX‐YY = age range. Pink data points represent females and blue data points represent males. Groups with statistically significant differences found in planned comparisons are denoted by an overhead bar and star (both here and in the supplement). Data points with a red cross enclosed are outliers greater than the third quartile plus 1.5 times the interquartile range (both here and in the data supplement). (a) No groups were found to have statistically significant differences when analysed by the planned comparisons. (b) Only one group was found to have statistically significant differences. (c) One further planned comparison was statistically significant that could not be represented in this plot: all healthy females vs. all healthy males. (d) One further planned comparison was statistically significant that cannot be represented in this plot: all healthy females vs. all healthy males.

The results from the one‐way ANOVA suggested that all metrics required further investigation with planned comparisons, as all p‐values were less than the critical value of 0.05. In the text and on the *x*‐axis of Figure 1 and figures in the Supplementary, comparisons between sex and age groups are abbreviated and should be read as such: F = female, M = male, H = healthy, U = unhealthy. The following four numbers XX‐YY, are the age brackets for each group, with XX being the lower age limit and YY being the upper age limit.

BCS had three statistically significant planned comparisons (Table S10: FH45‐54 vs MH45‐54, FH65‐74 vs MH65‐74 and all MH vs all FH all p≤ 0.001), these are shown in Figure 1c. MCS had a statistically significant result for MH65‐74 vs MU65‐74, see Figure 1b, and for FH45‐54 vs FH65‐74 (both p≤ 0.002). For GCS all MH vs all FH was statistically significant (p= 0.001, see Figure 1d). ACS had no planned comparisons that returned statistically significant results. Broadly these results suggest that only differences in basal and GCSs exist between the sexes, with no significant differences found for apical or MCS.

The volume metrics EDV, ESV and ESVi all had multiple instances of being statistically significant (see Table S11). Planned comparisons corresponding to the three age range comparisons between the healthy sexes were all statistically significant for EDV, ESV and ESVi (all p≤ 0.001), suggesting differences in volumes between the sexes (see Figures S1–S3). Additionally, for EDV, FH45‐54 vs FH55‐64, FH45‐54 vs FH65‐74, MH45‐54 vs MH65‐74, all MH vs all FH and all MU vs all FU were also statistically significant (all p≤ 0.003, see Table S11). MH45‐54 vs MH65‐74, MH65‐74 vs MU65‐74, all MH vs all FH were statistically significant for ESV (all p≤ 0.003). For ESVi, MH65‐74 vs MU65‐74 and all MH vs all FH were both also statistically significant (both p≤ 0.001). LVEF had only two comparisons that resulted in statistically significant differences, MH65‐74 vs MU65‐74 and all MH vs all FH (both p≤ 0.001, see Figure S5). BSA had multiple instances of comparisons being statistically significant; the three age range comparisons between the healthy sexes, MH45‐54 vs MH 65–74, all MH vs all FH and all MU vs all FU (all p≤ 0.001, see Figure S4).

A list of all statistically significant planned comparisons is shown in Table 3.

**TABLE 3:** List of all planned comparisons which had statistically significant results to p≤ 0.003

Comparison	Variable	*p*‐value
Female Healthy 45–54 vs Female Healthy 55–64	EDV	0.003
Female Healthy 45–54 vs Female Healthy 65–74	MCS	0.002
	EDV	0.002
Female Healthy 45–54 vs Male Healthy 45–54	BCS	0.001
	EDV	0.001
	ESV	0.001
	ESVi	0.001
	BSA	0.001
Female Healthy 55–64 vs Male Healthy 55–64	EDV	0.001
	ESV	0.001
	ESVi	0.001
	BSA	0.001
Female Healthy 65–74 vs Male Healthy 65–74	BCS	0.001
	EDV	0.001
	ESV	0.001
	ESVi	0.001
	BSA	0.001
Male Healthy 45–54 vs Male Healthy 65–74	EDV	0.001
	ESV	0.003
	BSA	0.001
Male Healthy 65–74 vs Male Unhealthy 65–74	MCS	0.001
	LVEF	0.001
	ESV	0.001
	ESVi	0.001
All Male Healthy vs All Female Healthy	BCS	0.001
	GCS	0.001
	LVEF	0.001
	EDV	0.001
	ESV	0.001
	ESVi	0.001
	BSA	0.001
All Female Unhealthy vs All Male Unhealthy	EDV	0.003
	BSA	0.001

#### 3.2.1 Correlations

For healthy males and females, all correlations for both EDV and ESV with both age and BSA were statistically significant, apart from the correlation of age and ESV for healthy females (all p≤ 0.05, Tables S14 and S12). Also for healthy males and females, correlations between MCS and age were found, and for males, a correlation of MCS with BSA was also found (all p≤ 0.05, Tables S14 and S12). Furthermore, a statistically significant correlation between age and ESVi for healthy males was found (all p= 0.028, Table S14). No further significant correlations between age and BSA were found for the healthy groups.

For the unhealthy cohorts, fewer statistically significant correlations were found. For unhealthy females, the only significant correlation found was between age and EDV (p= 0.039, Table S13). For unhealthy men, significant correlations were found between age and LVEF, and between BSA and EDV (both p≤ 0.41, Table S15). No other significant correlations between age and BSA were found for the unhealthy groups.

The only correlation where the bootstrapped 95% CIs suggested that statistical significance found by the correlation was incorrect was for unhealthy females between age and EDV (Table S13). This was most likely due to the low *n* for this group. All other 95% CIs corroborated the statistically significant p‐values for the correlations. Whilst the spread of the data allowed line fits to be made and statistically significant p‐values in several of the analyses, the Pearson correlation coefficients did not indicate that any of the correlations were strong, with Pearson *r* values ranging from −0.49 to 0.482. These data are detailed in Tables S12–S15.

## 4 DISCUSSION

The present study aimed to investigate clinically relevant age‐ and sex‐specific LV values for healthy and unhealthy Caucasian adults, derived from CMR. A cohort of 710 individuals taken from the UK Biobank population was used.

### 4.1 Male‐to‐female comparisons

Significant differences were found for all strain measures when comparing all males to all females, regardless of health status or age group (see Table 1). Comparing only all healthy males to all healthy females showed that BCS and GCS remained statistically different (both p≤ 0.001, Table S10), with males having lower absolute values of both BCS and GCS, indicating there may be a difference in function between the sexes, which, as discussed in more detail below, may reflect the fact that whilst volumes are larger for males, ejection fractions are lower. For age‐range paired comparisons significant differences were only found for BCS in the youngest and eldest groups (both p= 0.001, Table S10).

These results confirm previous work where differences in GCS were found between males and females, with some studies reporting statistically significant differences (Andre et al., 2015; Mangion et al., 2016), and one reporting differences, but not of significance (Augustine et al., 2013). Støylen et al. however, found no difference in endocardial strains but did find differences in epicardial strains (Støylen et al., 2019). These different conclusions may arise in part due to differences in the accuracy of the different imaging modalities used, as Mangion et al. found differences in strain using both 1.5 T and 3 T MRI (Mangion et al., 2016). Given that cardiovascular MR affords greater spatial and contrast resolution than echo, regardless of patient body habitus, it is more appropriate for our layer‐specific strain measure, which requires clear endocardial definition throughout the cardiac cycle. This higher endocardial resolution may explain why statistically significant differences in some endocardial strains were found here, but not in the study by Støylen et al. which used echocardiography.

Statistically significant differences were also found for all volumetric measures and BSA when comparing all males to all females, regardless of health or age (see Table 1). Statistically significant differences for EDV, ESV, ESVi and BSA were found between all four healthy male‐to‐female comparisons, with males having higher mean values than females (see Figures [Link], [Link]). These results were expected as males are larger than females on average and similar differences have been previously reported (Petersen et al., 2017). For LVEF, the only comparison found to be statistically significant was that between all healthy males to all healthy females with healthy males having a slightly lower LVEF on average (p= 0.001, Table S11), agreeing with a result found by Petersen et al. (ibid.). This lowered LVEF accords with the lower average strain values for males which, when coupled with the larger ventricular volumes for males, appear to create sufficient blood flow in males.

### 4.2 Healthy age group comparisons

The only strain metric to have any significant differences when comparing within‐sex age ranges for healthy populations was MCS, it was found to be significant for the comparison between the youngest and oldest healthy female groups (p= 0.002, Table S10).

MCS was also the only strain metric which showed statistically significant correlations to either age or BSA for healthy groups. A significant correlation was found between MCS in healthy females and age (Table S12) and also between MCS in healthy males and both age and BSA (Table S14). Two previous studies also found no correlation between global endocardial circumferential strain (Andre et al., 2015; Støylen et al., 2019), though Mangion et al. (2016) did find correlations relating global strains to age. The mean ages of the cohorts used in these three studies were all different and all lower than in the present study, preventing a like‐for‐like comparison. In addition, the circumferential strain measurement technique used by Støylen et al. (2019) was mid‐wall only and not a global measure. Mid‐wall circumferential strain is thought to be most closely related to mean GCS and often used in its place for reasons of speed and ease (Lee et al., 2019; Støylen et al., 2019). The study by Støylen et al. (2019) also derived circumferential strains from geometrical considerations combined with LV blood pool diameters, likely a less accurate method than the MR‐derived layer specific method of this study that uses endocardial‐perimeter lengths. Given that only MCS was found to vary significantly, it is possible that when the underlying change is included within the GCS calculation it is obscured by noise or is too small to discern in a statistically significant manner.

The significant MCS correlations with age for both females and males, and the significant result for healthy females aged 45–54 versus those aged 65–74 potentially indicate a change in function with age, to accompany the changes seen with the volumetric measures EDV, ESV and ESVi. Sex‐ and age‐specific patterns of LV remodelling have been reported by Hung et al., with females exhibiting pronounced changes in LV torsion, and a tendency towards greater LV concentricity (Hung et al., 2017), which may relate to the positive correlation of MSC with age.

There were statistically significant differences between age groups for healthy males. EDV and ESV for healthy males aged 45–54 versus 65–75 were significantly different (both p≤ 0.003, see Table S11). EDV, for healthy males aged 45–54 versus 55–64 was just above the threshold for statistical significance (p= 0.005; Table S11 respectively) matching the results reported by Petersen et al. (2017). Overall, these results for ventricular volumes align with the findings in Petersen et al. that both absolute and indexed measures of LV EDVs and stroke volumes were lower with increasing age. For ESVi and LVEF, no differences between age groups for healthy males were found.

Differences in EDV were significant between healthy females aged 45–54 and both the 55–64 and 65–74 groups (both p≤ 0.003, Table S11), agreeing with findings reported by Petersen et al., who also found that both absolute and indexed measures of EDV, ESV and stroke volume were smaller with increasing age in females. For ESV, ESVi and LVEF, no differences between age groups for healthy females were found.

There were statistically significant, but weak correlations between age and EDV for both healthy males and females (Tables S12 and S14). There was also a statistically significant correlation between ESV and age in healthy males (r= −0.192, p= 0.001, Table S14), whilst for females the relationship was almost significant (r= −0.120, p= 0.054, Table S12). Petersen et al. (2017) found similar correlations. The BSA correlation results also suggest that the data is following the same trends reported by Petersen et al., with EDV and ESV both increasing with BSA (Tables S12 and S14).

### 4.3 Healthy to unhealthy population comparison

In comparing all healthy participants to all unhealthy participants (irrespective of sex or age), all strain measures were found to be statistically significant (see Table 1). This suggests an overall difference in strain between the two populations, with healthy hearts exhibiting larger strain magnitudes than unhealthy hearts, in agreement with studies showing strains may be useful in predicting outcomes following MI (Gavara et al., 2017; Koos et al., 2013; Moen et al., 2011; Rademakers & Nagel, 2018). In addition, all volumetric measures and BSA were also found to be statistically significant (see Table 1), with unhealthy participants having larger hearts (larger ESV and EDV) and smaller LVEF when comparing all healthy with all unhealthy participants. Taken together these results suggest a link between morphological and functional changes occurring due to disease. It is likely that diseased myocardium that has underdone negative remodelling and ventricular dilatation, leading to larger end‐diastolic volumes, is then unable to generate sufficient stress through myocardial contraction to overcome the higher resultant fluid loading. This means strain magnitudes will be lower than in healthy hearts, which from geometric considerations implies a smaller stroke volume and LVEF.

When comparing strains in age and sex‐matched healthy and unhealthy groups, the only statistically significant difference was for MCS in males aged 65–74 (p= 0.001, Table S10), with strains lower in magnitude for the unhealthy group. In addition to MCS, several volumetric measures (LVEF, ESV and ESVi) and BSA were also significantly different between healthy and unhealthy males 65–74, with LVEF lower, but ESV and ESVi larger, in the unhealthy group, thus explaining the lowered LVEF. However, there were no significant differences between any volume metrics for any other matched groups.

### 4.4 Links between MI and strains

In Mansell et al. (2021), strain metrics were shown to be sensitive to MI location in the acute and early‐chronic phases post‐MI. Unfortunately, whilst strains do appear to indicate a difference between healthy and unhealthy heart function, such detailed links between MI and strains are hard to draw in this study for several reasons. Firstly, the time since the cardiac event in the previous study was in the order of days, as opposed to years, and the disease status of the unhealthy UK Biobank cohort was more complex than in the animal experiments, comprising those having suffered STEMI, NSTEMI and HF. Secondly, the precise location of the infarct was unknown in the UK Biobank data, meaning any correlation between infarct location and regional strain could not be determined. To definitively test the ability of the strain metric to localise myocardial infarcts in humans a targeted study with appropriate patient recruitment would be required.

### 4.5 Limitations

Despite more than 12,000 UK Biobank participants fulfilling the inclusion criteria, sample sizes for the unhealthy cohorts, particularly in the lower age ranges, remained small, affecting both the power of the statistics and statistical analyses. The disease state of these unhealthy groups was also heterogeneous due to varying elapsed time since the cardiac event and possible medical interventions, though all unhealthy participants were still reasonably healthy. This limitation may be difficult to overcome in future studies due to the heavily skewed prevalence of MI with increasing age (Dhingra & Vasan, 2012; Rodgers et al., 2019). Furthermore, all members of the unhealthy cohorts were also survivors of MI and HF, potentially introducing a survivorship bias into the data, as those with potentially worse cases of MI and HF would not be included.

In Mansell et al. (2021), strain metrics were shown to be sensitive to MI location in the acute and early‐chronic phases post‐MI. Unfortunately, such detailed links could not be assessed in this study since the length of time since MI was of the order of years, the precise location of the infarct was unknown and the varying diagnoses (STEMI, NSTEMI and HF) indicate a range of infarct severities, including the possibility of no MI in some cases of HF.

Finally, a BMI of up to 30 $\mathrm{kg}/m^{2}$ was used as one of the inclusion criteria. Whilst BMIs at the top of this range are technically considered to be overweight, and obesity has been shown to affect cardiac structure and function in an otherwise healthy population (Rider et al., 2009, 2011), other studies have also used these same inclusion criteria based on the fact that this BMI represents a ‘new normal’ for the population, with 58% of females and 65% of males in the UK having a BMI of more than 25 $\mathrm{kg}/m^{2}$ in 2014 (HSCIC, 2016; Petersen et al., 2017; Støylen et al., 2019). Thus, we argue, that those with a BMI of up to 30 $\mathrm{kg}/m^{2}$ should be included when statistics on the average healthy population are required.

## 5 CONCLUSIONS

The aim of this study was to characterise changes in LV standard clinical and strain metrics in both sexes, and to identify changes between healthy and unhealthy groups for both sexes, using imaging data from the UK Biobank. The main findings were that LV function as measured by basal strain and GCSs differed between healthy males and females, with males having lower absolute values of these strains. Furthermore, both functional and morphological variables differed between healthy and unhealthy participants, particularly males aged 65–74, which showed increased MCS, reduced LVEF and increased ESV and ESVi, in that unhealthy group. Finally, some data suggested that MCS may correlate positively with age, but additional data is required to confirm this.

These results suggest that if regional circumferential strains are to be used clinically for assessing cardiovascular health, due care must be taken to ensure critical values are indexed correctly to age and sex, whilst also validating the measurements against different imaging modalities, scanners, strain methods and research centres. Future studies should also investigate whether changes in strain values are observed for other cardiovascular diseases such as myocarditis and early‐stage heart failure. Finally, as these results apply only to British and Irish white adults, future work should study larger cohorts of age‐ranged and specific ethnic groups to assess whether these conclusions apply more generally and to determine the corresponding population‐level statistics.

## Supporting information


Figure S1

Figure S2

Figure S3

Figure S4

Figure S5

Appendix S1

Table S3

## Data Availability

The raw data for this study is available currently to registered users of the UK Biobank (https://www.ukbiobank.ac.uk/). Restrictions apply to the availability of these data, which were used under license for this study. The data generated that support the findings of this study will be returned to the UK Biobank, which may then make them available to registered users.
