# Supplementary material for: MRI‐based strain measurements reflect morphological changes following myocardial infarction: A study on the UK Biobank cohort
Source: J Anat. 2022 Dec 9;242(1):102–11. doi: 10.1111/joa.13787 (PMC9773168; doi:10.1111/joa.13787)
Supplement: Supplementary file 6 — Appendix S1 [file JOA-242-102-s003.docx]

# Online Supplementary Materials

Table 1: Results from Student’s t-test for all males vs. all females, as well as all healthy vs. all unhealthy participants. All results were statistically significant.

| Variable | All Males vs All Females *p*-values | All Healthy vs All Unhealthy *p*-values |
| --- | --- | --- |
| ESV | 0*.*001 | 0*.*001 |
| EDV | 0*.*001 | 0*.*001 |
| LVEF | 0*.*001 | 0*.*001 |
| ESVi | 0*.*001 | 0*.*001 |
| BSA | 0*.*001 | 0.005 |
| ACS | 0.002 | 0.006 |
| MCS | 0.003 | 0.001 |
| BCS | 0*.*001 | 0*.*001 |
| GCS | 0*.*001 | 0*.*001 |

Table 2: Further data for the Student’s t-test for all male vs. all female volunteers. Where the Variance *p*-value is less than 0.05, the data shown assumes that the variances between the groups was not equal.

| Variables | Variance *p*-value | Mean  Difference | Std. Error  Difference | 95% Confidence Interval of the  Difference | |
| --- | --- | --- | --- | --- | --- |
|  |  |  |  | Lower | Upper |
| ESV | 0 | -22.5 | 1.3 | -25.2 | -19.9 |
| EDV | 0 | -38.9 | 2.1 | -43.0 | -34.9 |
| LVEF | 0.002 | 3.3 | 0.5 | 2.4 | 4.2 |
| ESVi | 0 | -7.3 | 0.7 | -8.7 | -6.0 |
| BSA | 0.06 | -0.3 | 0.01 | -0.3 | -0.2 |
| ACS | 0.03 | -2.1 | 0.7 | -3.4 | -0.8 |
| MCS | 0 | -1.1 | 0.4 | -1.8 | -0.4 |
| BCS | 0.2 | -2.6 | 0.4 | -3.4 | -1.8 |
| GCS | 0.001 | -1.8 | 0.4 | -2.6 | -1.1 |

Table 3: Further data for the Student’s t-test for all healthy vs. all unhealthy volunteers. Where the Variance *p*-value is less than 0.05, the data shown assumes that the variances between the groups was not equal.

| Variables | Variance *p*-value | Mean  Difference | Std. Error  Difference | 95% Confidence Interval of the  Difference | |
| --- | --- | --- | --- | --- | --- |
|  |  |  |  | Lower | Upper |
| ESV | 0 | -17.9 | 2.8 | -23.4 | -12.4 |
| EDV | 0.07 | -18.3 | 3.3 | -24.9 | -11.8 |
| LVEF | 0 | 5.4 | 0.8 | 3.9 | 6.9 |
| ESVi | 0 | -8.7 | 1.4 | -11.4 | -6.0 |
| BSA | 0.003 | -0.05 | 0.02 | -0.08 | -0.01 |
| ACS | 0 | -3.5 | 1.2 | -5.9 | -1.0 |
| MCS | 0 | -2.1 | 0.6 | -3.4 | -0.9 |
| BCS | 0 | -2.3 | 0.7 | -3.7 | -1.0 |
| GCS | 0 | -2.8 | 0.7 | -4.2 | -1.4 |

Table 4: Results from the initial one-way ANOVAs. All *p*-values were less than the required 0.05.

| ANOVA | *p*-value |
| --- | --- |
| ESV | 0.001 |
| EDV | 0.001 |
| LVEF | 0.001 |
| ESVi | 0.001 |
| BSA | 0.001 |
| ACS | 0.019 |
| MCS | 0.001 |
| BCS | 0.001 |
| GCS | 0.001 |

Table 5: Baseline characteristics for healthy females of all ages.

| Measure | *n* | Mean | Median | St. Dev. | Lower 95% CI | Upper 95% CI |
| --- | --- | --- | --- | --- | --- | --- |
| BMI (*kg/m*^2^) | 281 | 24.53 | 24.38 | 2.78 | 24.2 | 24.86 |
| HR (bpm) | 260 | 62.09 | 61 | 9.22 | 60.95 | 63.23 |
| Weight (kg) | 281 | 65.51 | 64.7 | 8.34 | 64.51 | 66.51 |
| Age (years) | 281 | 60.17 | 61 | 7.58 | 59.27 | 61.07 |
| ESV (mL) | 260 | 52.92 | 52 | 11.6 | 51.49 | 54.35 |
| EDV (mL) | 260 | 124.2 | 124 | 21.2 | 121.5 | 126.8 |
| LVEF (%) | 260 | 57.42 | 58 | 4.91 | 56.81 | 58.03 |
| ESVi (*mL/m*^2^) | 260 | 31.01 | 30.09 | 6.4 | 30.22 | 31.8 |
| BSA (*m*^2^) | 260 | 1.71 | 1.7 | 0.12 | 1.7 | 1.72 |
| ACS (%) | 281 | -42.43 | -42.17 | 7.68 | -43.35 | -41.51 |
| MCS (%) | 281 | -29.68 | -29.74 | 3.9 | -30.15 | -29.21 |
| BCS (%) | 281 | -32.8 | -32.42 | 5.01 | -33.4 | -32.2 |
| GCS (%) | 281 | -34.29 | -34.4 | 4.09 | -34.78 | -33.8 |

Table 6: Baseline characteristics for unhealthy females of all ages.

| Measure | *n* | Mean | Median | St. Dev. | Lower 95% CI | Upper 95% CI |
| --- | --- | --- | --- | --- | --- | --- |
| BMI (*kg/m*^2^) | 20 | 24.85 | 24.73 | 2.84 | 23.58 | 26.12 |
| HR (bpm) | 18 | 61.56 | 58 | 10.9 | 56.43 | 66.69 |
| Weight (kg) | 20 | 63.6 | 64.25 | 7.28 | 60.34 | 66.86 |
| Age (years) | 20 | 66.85 | 68.5 | 5.03 | 64.6 | 69.1 |
| ESV (mL) | 18 | 58.11 | 57 | 17.7 | 49.75 | 66.47 |
| EDV (mL) | 18 | 126.6 | 129 | 24 | 115.3 | 137.9 |
| LVEF (%) | 18 | 54.56 | 56 | 8.18 | 50.7 | 58.42 |
| ESVi (*mL/m*^2^) | 18 | 34.66 | 34.13 | 10.5 | 29.73 | 39.59 |
| BSA (*m*^2^) | 18 | 1.67 | 1.69 | 0.1 | 1.62 | 1.72 |
| ACS (%) | 20 | -39.97 | -42.74 | 13.9 | -46.18 | -33.76 |
| MCS (%) | 20 | -27.98 | -29.15 | 5.44 | -30.41 | -25.55 |
| BCS (%) | 20 | -30.94 | -30.64 | 7.16 | -34.14 | -27.74 |
| GCS (%) | 20 | -32.13 | -33.26 | 7.65 | -35.55 | -28.71 |

Table 7: Baseline characteristics for healthy males of all ages.

| Measure | *n* | Mean | Median | St. Dev. | Lower 95% CI | Upper 95% CI |
| --- | --- | --- | --- | --- | --- | --- |
| BMI (*kg/m*^2^) | 293 | 25.65 | 25.9 | 2.42 | 25.37 | 25.93 |
| HR (bpm) | 274 | 60.32 | 60 | 8.86 | 59.25 | 61.39 |
| Weight (kg) | 293 | 80.45 | 80.4 | 9.5 | 79.34 | 81.56 |
| Age (years) | 293 | 60.35 | 60 | 7.87 | 59.43 | 61.27 |
| ESV (mL) | 274 | 72.4 | 70 | 16.8 | 70.37 | 74.43 |
| EDV (mL) | 274 | 161.4 | 159 | 29 | 157.9 | 165 |
| LVEF (%) | 274 | 55.3 | 56 | 5.59 | 54.62 | 55.98 |
| ESVi (*mL/m*^2^) | 274 | 36.56 | 35.98 | 7.85 | 35.61 | 37.51 |
| BSA (*m*^2^) | 274 | 1.98 | 1.97 | 0.14 | 1.96 | 2 |
| ACS (%) | 293 | -41.08 | -40.86 | 7.9 | -42 | -40.16 |
| MCS (%) | 293 | -29.08 | -28.67 | 4.35 | -29.59 | -28.57 |
| BCS (%) | 293 | -30.53 | -30.73 | 3.89 | -30.98 | -30.08 |
| GCS (%) | 293 | -32.99 | -32.99 | 4.44 | -33.51 | -32.47 |

Table 8: Baseline characteristics for unhealthy males of all ages.

| Measure | *n* | Mean | Median | St. Dev. | Lower 95% CI | Upper 95% CI |
| --- | --- | --- | --- | --- | --- | --- |
| BMI (*kg/m*^2^) | 111 | 25.68 | 25.78 | 2.28 | 25.25 | 26.11 |
| HR (bpm) | 101 | 56.02 | 54 | 9.04 | 54.22 | 57.82 |
| Weight (kg) | 111 | 78.19 | 78.3 | 8.65 | 76.55 | 79.83 |
| Age (years) | 111 | 66.8 | 68 | 5.36 | 65.78 | 67.82 |
| ESV (mL) | 101 | 84.88 | 78 | 28.8 | 79.14 | 90.62 |
| EDV (mL) | 101 | 167.9 | 161 | 37 | 160.5 | 175.2 |
| LVEF (%) | 101 | 50.25 | 51 | 7.68 | 48.72 | 51.78 |
| ESVi (*mL/m*^2^) | 101 | 44 | 40.51 | 14.8 | 41.06 | 46.94 |
| BSA (*m*^2^) | 101 | 1.93 | 1.93 | 0.13 | 1.9 | 1.96 |
| ACS (%) | 111 | -37.97 | -41.54 | 13.8 | -40.6 | -35.34 |
| MCS (%) | 111 | -27.1 | -27.81 | 7.4 | -28.5 | -25.7 |
| BCS (%) | 111 | -29 | -28.28 | 7.45 | -30.41 | -27.59 |
| GCS (%) | 111 | -30.61 | -32.14 | 7.75 | -32.08 | -29.14 |

Table 9: Mean time in years since initial cardiac event. * There was only one participant in this group.

| Unhealthy  Group | Mean time  (years) |
| --- | --- |
| Females 45-54 | 14.7* |
| Females 55-64 | 2.8 |
| Females 65-74 | 4.3 |
| Males 45-54 | 4.6 |
| Males 55-64 | 5.9 |
| Males 65-74 | 7.7 |

| Table10:  *P*  valuesforallstrainandstrainratemetrics.Resultshighlightedingreenwithboldtextand*,  -  indicatesresultswhicharestatisticallysignificantto  *p*  ≤  0.003. | \| GCS \| 0.439 \| 0.226 \| 0.669 \| 0.066 \| 0.051 \| 0.015 \| 0.682 \| 0.824 \| 0.865 \| 0.01 \| 0.311 \| 0.303 \| 0.569 \| **0.001*** \| 0.283 \| 0.228 \| 0.877 \| \| --- \| --- \| --- \| --- \| --- \| --- \| --- \| --- \| --- \| --- \| --- \| --- \| --- \| --- \| --- \| --- \| --- \| --- \| \| BCS \| 0.462 \| 0.785 \| 0.245 \| **0.001*** \| 0.004 \| **0.001*** \| 0.505 \| 0.709 \| 0.762 \| 0.118 \| 0.266 \| 0.147 \| 0.857 \| **0.001*** \| 0.305 \| 0.207 \| 0.288 \| \| MCS \| 0.041 \| **0.002*** \| 0.272 \| 0.569 \| 0.376 \| 0.153 \| 0.098 \| 0.069 \| 0.763 \| **0.001*** \| 0.145 \| 0.563 \| 0.369 \| 0.061 \| 0.248 \| 0.513 \| 0.719 \| \| ACS \| 0.556 \| 0.816 \| 0.412 \| 0.321 \| 0.078 \| 0.449 \| 0.839 \| 0.969 \| 0.860 \| 0.033 \| 0.725 \| 0.790 \| 0.436 \| 0.060 \| 0.437 \| 0.299 \| 0.948 \| \| Planned  Comparison \| FemaleHealthy45-54vs.FemaleHealthy55-64 \| FemaleHealthy45-54vs.FemaleHealthy65-74 \| FemaleHealthy55-64vs.FemaleHealthy65-74 \| FemaleHealthy45-54vs.MaleHealthy45-54 \| FemaleHealthy55-64vs.MaleHealthy55-64 \| FemaleHealthy65-74vs.MaleHealthy65-74 \| MaleHealthy45-54vs.MaleHealthy55-64 \| MaleHealthy45-54vs.MaleHealthy65-74 \| MaleHealthy55-64vs.MaleHealthy65-74 \| MaleHealthy65-74vs.MaleUnhealthy65-74 \| FemaleHealthy65-74vs.FemaleUnhealthy65-74 \| MaleHealthy55-64vs.MaleUnhealthy55-64 \| MaleUnhealthy55-64vs.MaleUnhealthy65-74 \| AllMalesHealthyvs.AllFemalesHealthy \| AllFemalesHealthyvs.AllFemalesUnhealthy \| AllMalesHealthyvs.AllMalesUnhealthy \| AllFemalesUnhealthyvs.AllMalesUnhealthy \| |
| --- | --- | --- | --- | --- | --- | --- | --- | --- | --- | --- | --- | --- | --- | --- | --- | --- | --- | --- | --- | --- | --- | --- | --- | --- | --- | --- | --- | --- | --- | --- | --- | --- | --- | --- | --- | --- | --- | --- | --- | --- | --- | --- | --- | --- | --- | --- | --- | --- | --- | --- | --- | --- | --- | --- | --- | --- | --- | --- | --- | --- | --- | --- | --- | --- | --- | --- | --- | --- | --- | --- | --- | --- | --- | --- | --- | --- | --- | --- | --- | --- | --- | --- | --- | --- | --- | --- | --- | --- | --- | --- | --- |

| Table11:  *P*  valuesforallstrainandstrainratemetrics.Resultshighlightedingreenwithboldtextand*,  -  indicatesresultswhicharestatisticallysignificantto  *p*  ≤  0.003. | \| BSA \| 0.196 \| 0.315 \| 0.843 \| **0.001*** \| **0.001*** \| **0.001*** \| 0.048 \| **0.001*** \| 0.071 \| 0.886 \| 0.074 \| 0.111 \| 0.701 \| **0.001*** \| 0.574 \| 0.004 \| **0.001*** \| \| --- \| --- \| --- \| --- \| --- \| --- \| --- \| --- \| --- \| --- \| --- \| --- \| --- \| --- \| --- \| --- \| --- \| --- \| \| ESVi \| 0.010 \| 0.040 \| 0.781 \| **0.001*** \| **0.001*** \| **0.001*** \| 0.053 \| 0.039 \| 0.701 \| **0.001*** \| 0.346 \| 0.008 \| 0.632 \| **0.001*** \| 0.206 \| 0.052 \| 0.090 \| \| ESV \| 0.004 \| 0.024 \| 0.711 \| **0.001*** \| **0.001*** \| **0.001*** \| 0.150 \| **0.003*** \| 0.392 \| **0.001*** \| 0.499 \| 0.013 \| 0.734 \| **0.001*** \| 0.209 \| 0.074 \| 0.012 \| \| EDV \| **0.003*** \| **0.002*** \| 0.693 \| **0.001*** \| **0.001*** \| **0.001*** \| 0.005 \| **1.00E-03*** \| 0.070 \| 0.018 \| 0.962 \| 0.008 \| 0.094 \| **0.001*** \| 0.248 \| 0.151 \| **0.003*** \| \| LVEF \| 0.252 \| 0.872 \| 0.209 \| 0.020 \| 0.005 \| 0.017 \| 0.755 \| 0.516 \| 0.357 \| **0.001*** \| 0.351 \| 0.031 \| 0.485 \| **0.001*** \| 0.202 \| 0.044 \| 0.263 \| \| Planned  Comparison \| FemaleHealthy45-54vs.FemaleHealthy55-64 \| FemaleHealthy45-54vs.FemaleHealthy65-74 \| FemaleHealthy55-64vs.FemaleHealthy65-74 \| FemaleHealthy45-54vs.MaleHealthy45-54 \| FemaleHealthy55-64vs.MaleHealthy55-64 \| FemaleHealthy65-74vs.MaleHealthy65-74 \| MaleHealthy45-54vs.MaleHealthy55-64 \| MaleHealthy45-54vs.MaleHealthy65-74 \| MaleHealthy55-64vs.MaleHealthy65-74 \| MaleHealthy65-74vs.MaleUnhealthy65-74 \| FemaleHealthy65-74vs.FemaleUnhealthy65-74 \| MaleHealthy55-64vs.MaleUnhealthy55-64 \| MaleUnhealthy55-64vs.MaleUnhealthy65-74 \| AllMalesHealthyvs.AllFemalesHealthy \| AllFemalesHealthyvs.AllFemalesUnhealthy \| AllMalesHealthyvs.AllMalesUnhealthy \| AllFemalesUnhealthyvs.AllMalesUnhealthy \| |
| --- | --- | --- | --- | --- | --- | --- | --- | --- | --- | --- | --- | --- | --- | --- | --- | --- | --- | --- | --- | --- | --- | --- | --- | --- | --- | --- | --- | --- | --- | --- | --- | --- | --- | --- | --- | --- | --- | --- | --- | --- | --- | --- | --- | --- | --- | --- | --- | --- | --- | --- | --- | --- | --- | --- | --- | --- | --- | --- | --- | --- | --- | --- | --- | --- | --- | --- | --- | --- | --- | --- | --- | --- | --- | --- | --- | --- | --- | --- | --- | --- | --- | --- | --- | --- | --- | --- | --- | --- | --- | --- | --- | --- | --- | --- | --- | --- | --- | --- | --- | --- | --- | --- | --- | --- | --- | --- | --- | --- | --- |

Table 12: Age and BSA correlations with other variables of interest for all healthy females. Statistically significant *p*-values are highlighted in green. The 95% CIs are taken from the bootstrapping analysis.

| Variable | Age Correlations | | | | |
| --- | --- | --- | --- | --- | --- |
|  | *n* | Pearson | *p*-value | 95% CIs | |
|  |  |  |  | Lower | Upper |
| EDV | 260 | -0.193 | 0.002 | -0.303 | -0.069 |
| ESV | 260 | -0.120 | 0.054 | -0.243 | -0.003 |
| LVEF | 260 | -0.059 | 0.344 | -0.189 | 0.068 |
| ESVi | 260 | -0.120 | 0.054 | -0.242 | 0.008 |
| ACS | 281 | 0.046 | 0.442 | -0.089 | 0.175 |
| MCS | 281 | -0.163 | 0.006 | -0.272 | -0.029 |
| BCS | 281 | -0.006 | 0.918 | -0.114 | 0.134 |
| GCS | 281 | -0.041 | 0.490 | -0.156 | 0.090 |
| Variable | BSA Correlations | | | | |
| EDV | 260 | 0.443 | 0.000 | 0.341 | 0.542 |
| ESV | 260 | 0.318 | 0.000 | 0.202 | 0.425 |
| LVEF | 260 | 0.065 | 0.300 | -0.070 | 0.190 |
| ESVi | 260 | -0.017 | 0.786 | -0.137 | 0.109 |
| ACS | 260 | -0.046 | 0.459 | -0.165 | 0.074 |
| MCS | 260 | -0.063 | 0.308 | -0.208 | 0.071 |
| BCS | 260 | 0.054 | 0.384 | -0.065 | 0.153 |
| GCS | 260 | -0.007 | 0.917 | -0.136 | 0.117 |

Table 13: Age and BSA correlations with other variables of interest for all unhealthy females. Statistically significant *p*-values are highlighted in green. The 95% CIs are taken from the bootstrapping analysis.

| Variable | Age Correlations | | | | |
| --- | --- | --- | --- | --- | --- |
|  | *n* | Pearson | *p*-value | 95% CIs | |
|  |  |  |  | Lower | Upper |
| EDV | 18 | -0.49 | 0.039 | -0.790 | 0.012 |
| ESV | 18 | -0.319 | 0.196 | -0.243 | -0.003 |
| LVEF | 18 | 0.005 | 0.984 | -0.517 | 0.672 |
| ESVi | 18 | -0.269 | 0.281 | -0.781 | 0.351 |
| ACS | 20 | -0.187 | 0.431 | -0.682 | 0.390 |
| MCS | 20 | -0.165 | 0.487 | -0.613 | 0.365 |
| BCS | 20 | 0.093 | 0.697 | -0.149 | 0.506 |
| GCS | 20 | -0.123 | 0.606 | -0.567 | 0.370 |
| Variable | BSA Correlations | | | | |
| EDV | 18 | 0.428 | 0.076 | -0.148 | 0.721 |
| ESV | 18 | 0.307 | 0.216 | 0.202 | 0.425 |
| LVEF | 18 | -0.155 | 0.538 | -0.594 | 0.526 |
| ESVi | 18 | 0.157 | 0.534 | -0.525 | 0.642 |
| ACS | 18 | 0.113 | 0.656 | -0.358 | 0.470 |
| MCS | 18 | 0.131 | 0.605 | -0.403 | 0.552 |
| BCS | 18 | 0.053 | 0.834 | -0.477 | 0.471 |
| GCS | 18 | 0.131 | 0.605 | -0.390 | 0.506 |

Table 14: Age and BSA correlations with other variables of interest for all healthy males. Statistically significant *p*-values are highlighted in green. The 95% CIs are taken from the bootstrapping analysis.

| Variable | Age Correlations | | | | |
| --- | --- | --- | --- | --- | --- |
|  | *n* | Pearson | *p*-value | 95% CIs | |
|  |  |  |  | Lower | Upper |
| EDV | 274 | -0.280 | 0.000 | -0.399 | -0.152 |
| ESV | 274 | -0.192 | 0.001 | -0.316 | -0.063 |
| LVEF | 274 | -0.047 | 0.439 | -0.171 | 0.082 |
| ESVi | 274 | -0.133 | 0.028 | -0.249 | -0.003 |
| ACS | 293 | -0.026 | 0.657 | -0.186 | 0.062 |
| MCS | 293 | -0.160 | 0.006 | -0.308 | -0.072 |
| BCS | 293 | -0.033 | 0.569 | -0.139 | 0.088 |
| GCS | 293 | -0.048 | 0.411 | -0.201 | 0.050 |
| Variable | BSA Correlations | | | | |
| EDV | 274 | 0.482 | 0.000 | 0.390 | 0.567 |
| ESV | 274 | 0.395 | 0.000 | 0.293 | 0.484 |
| LVEF | 274 | -0.049 | 0.420 | -0.168 | 0.070 |
| ESVi | 274 | 0.100 | 0.097 | -0.012 | 0.207 |
| ACS | 274 | 0.075 | 0.217 | -0.029 | 0.183 |
| MCS | 274 | 0.170 | 0.005 | 0.049 | 0.283 |
| BCS | 274 | 0.092 | 0.130 | -0.044 | 0.210 |
| GCS | 274 | 0.108 | 0.074 | -0.008 | 0.226 |

Table 15: Age and BSA correlations with other variables of interest for all unhealthy males. Statistically significant *p*-values are highlighted in green. The 95% CIs are taken from the bootstrapping analysis.

| Variable |  | Age Correlations | | | |
| --- | --- | --- | --- | --- | --- |
|  | *n* | Pearson | *p*-value | 95% CIs | |
|  |  |  |  | Lower | Upper |
| EDV | 101 | -0.114 | 0.256 | -0.315 | 0.065 |
| ESV | 101 | 0.049 | 0.626 | -0.159 | 0.200 |
| LVEF | 101 | -0.204 | 0.041 | -0.374 | -0.026 |
| ESVi | 101 | 0.048 | 0.632 | -0.151 | 0.215 |
| ACS | 111 | 0.089 | 0.351 | -0.049 | 0.339 |
| MCS | 111 | 0.148 | 0.120 | -0.064 | 0.342 |
| BCS | 111 | 0.020 | 0.835 | -0.131 | 0.192 |
| GCS | 111 | 0.101 | 0.293 | -0.056 | 0.320 |
| Variable |  | BSA Correlations | | | |
| EDV | 101 | 0.312 | 0.001 | 0.138 | 0.471 |
| ESV | 101 | 0.165 | 0.100 | -0.001 | 0.339 |
| LVEF | 101 | 0.074 | 0.465 | -0.107 | 0.266 |
| ESVi | 101 | -0.039 | 0.697 | -0.197 | 0.150 |
| ACS | 101 | -0.169 | 0.092 | -0.342 | 0.013 |
| MCS | 101 | -0.112 | 0.263 | -0.315 | 0.098 |
| BCS | 101 | -0.128 | 0.201 | -0.329 | 0.091 |
| GCS | 101 | -0.160 | 0.109 | -0.343 | 0.024 |

550


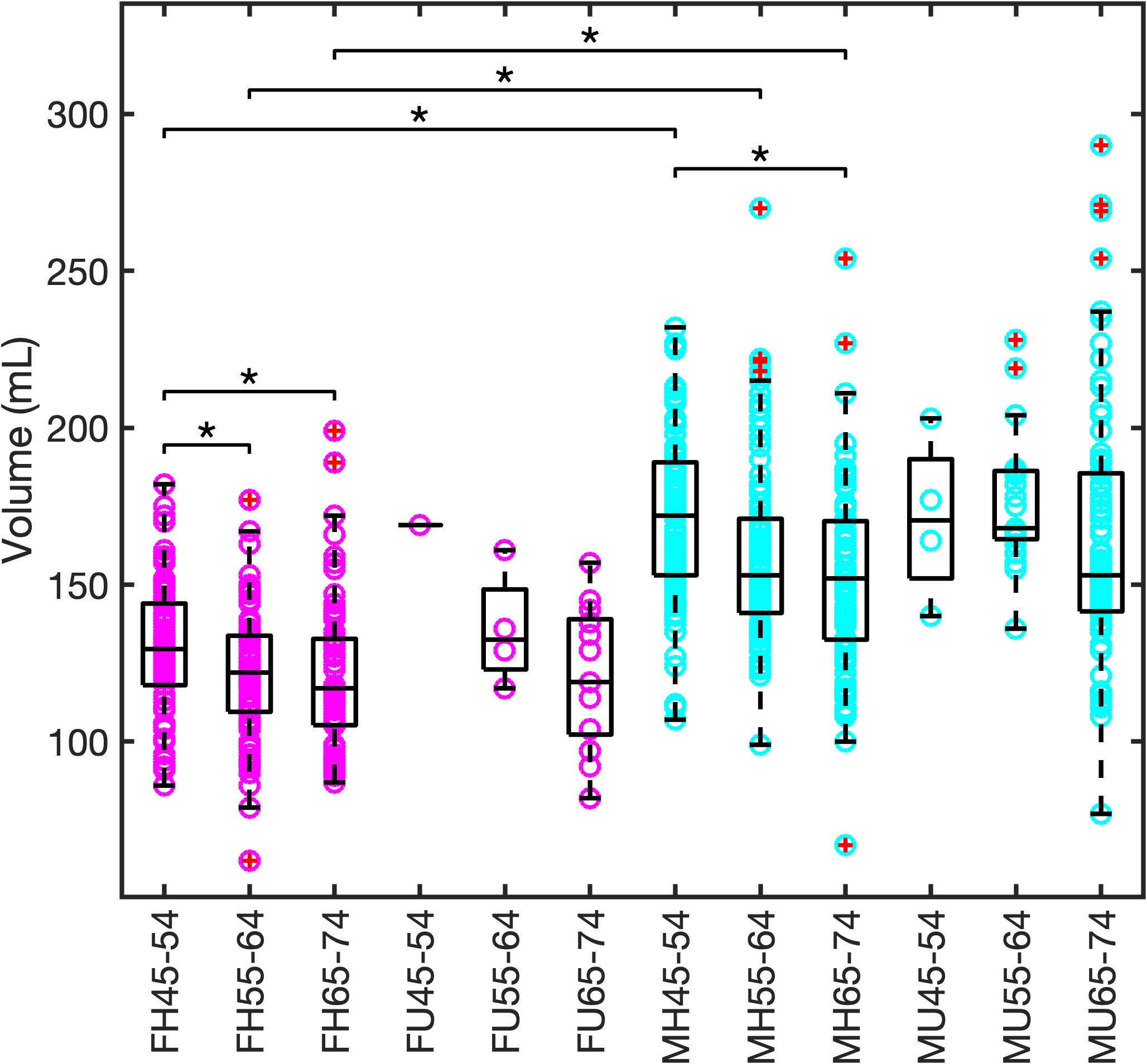


Figure 1: EDV boxplots for all groups: M = Male; F = Female; H = Healthy; U = Unhealthy; XX-YY = age range. Two further planned comparisons were statistically significant that could not be represented: all healthy females vs. all healthy males, and all unhealthy females vs. all unhealthy males.


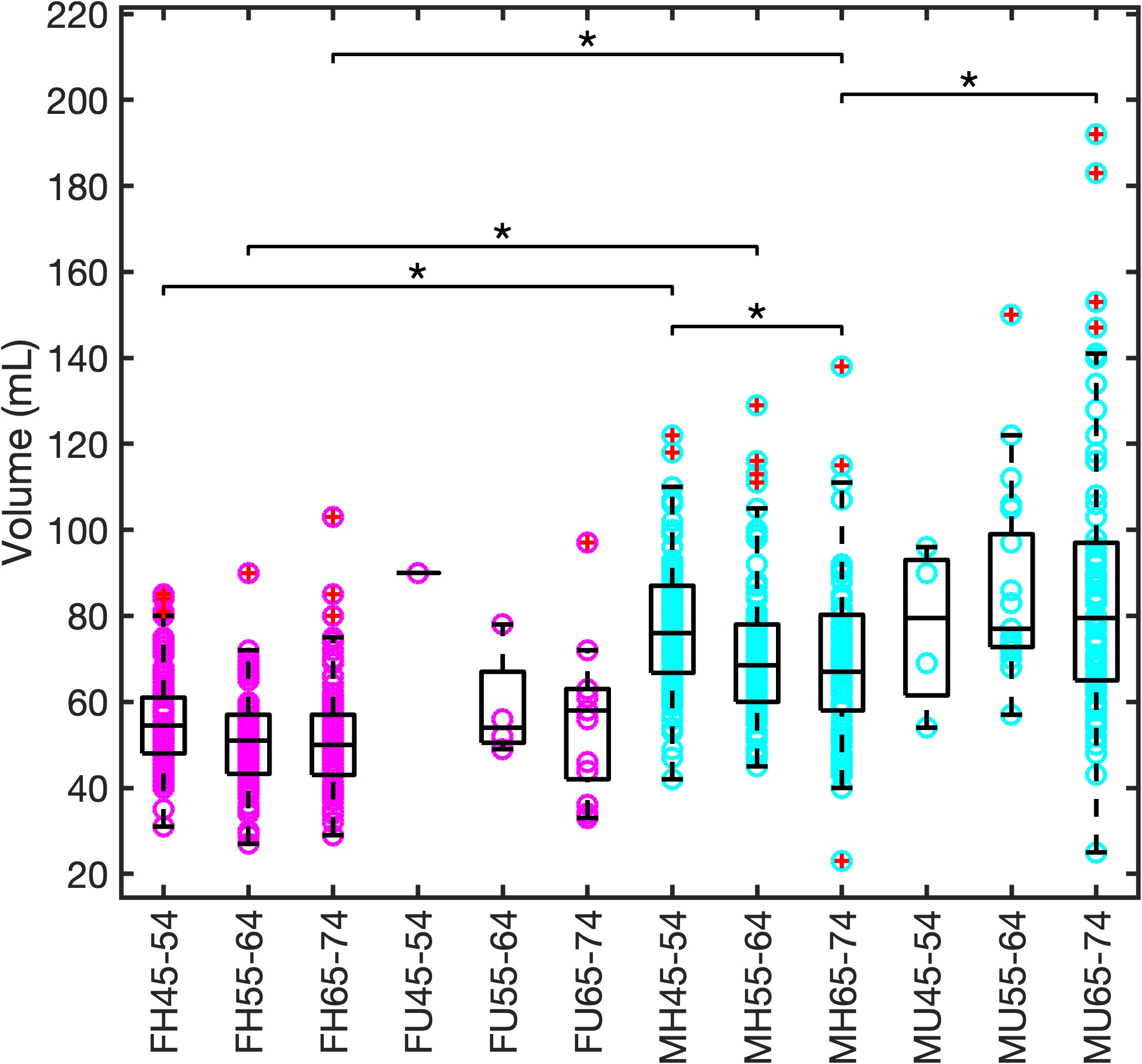


Figure 2: ESV boxplots for all groups: M = Male; F = Female; H = Healthy; U = Unhealthy; XX-YY = age range. One further planned comparison was statistically significant that could not be represented on this plot: all healthy females vs. all healthy males.


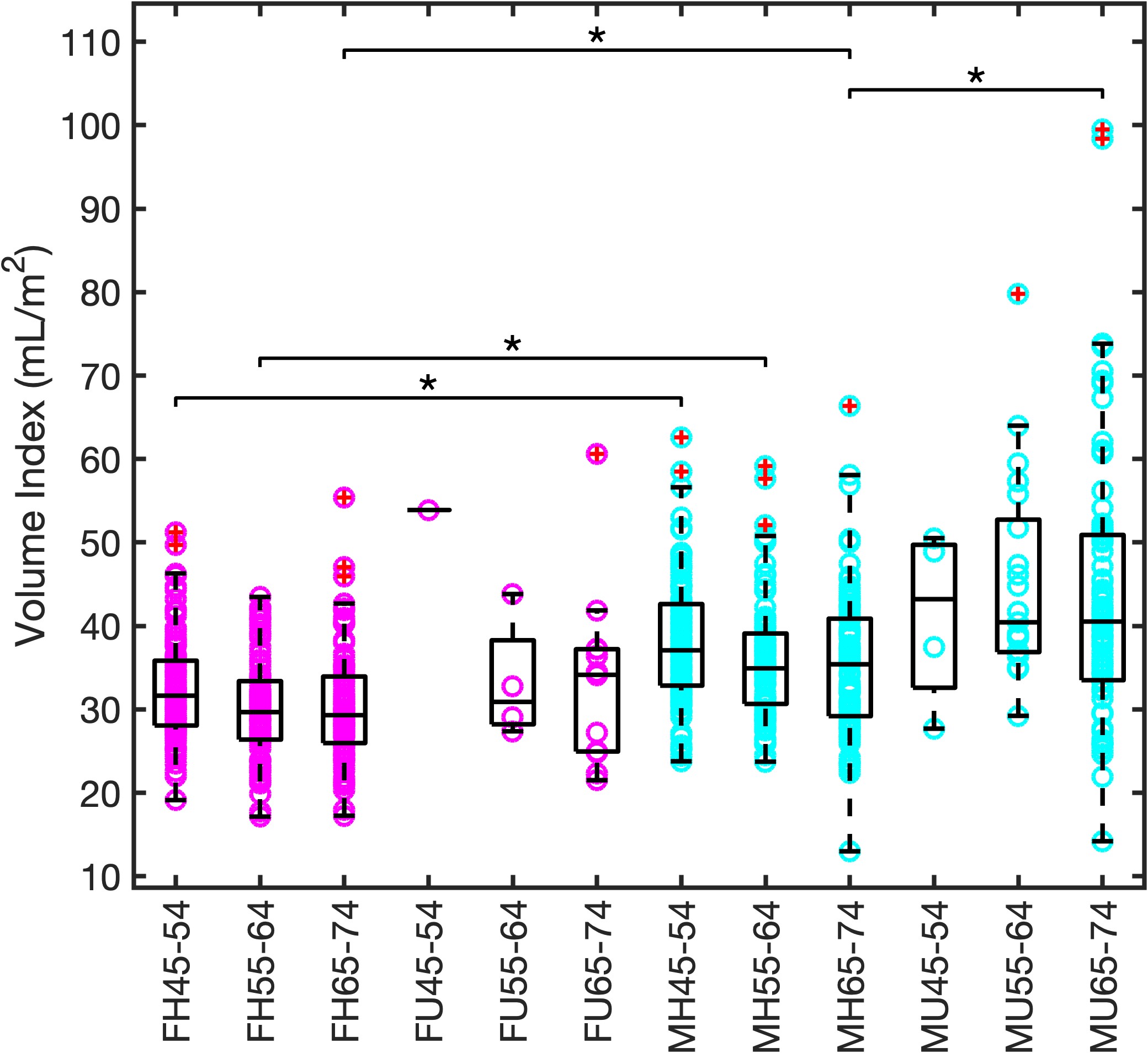


Figure 3: ESVi as boxplots for all groups: M = Male; F = Female; H = Healthy; U = Unhealthy; XX-YY = age range. One further planned comparison was statistically significant that could not be represented on this plot: all healthy females vs. all healthy males.


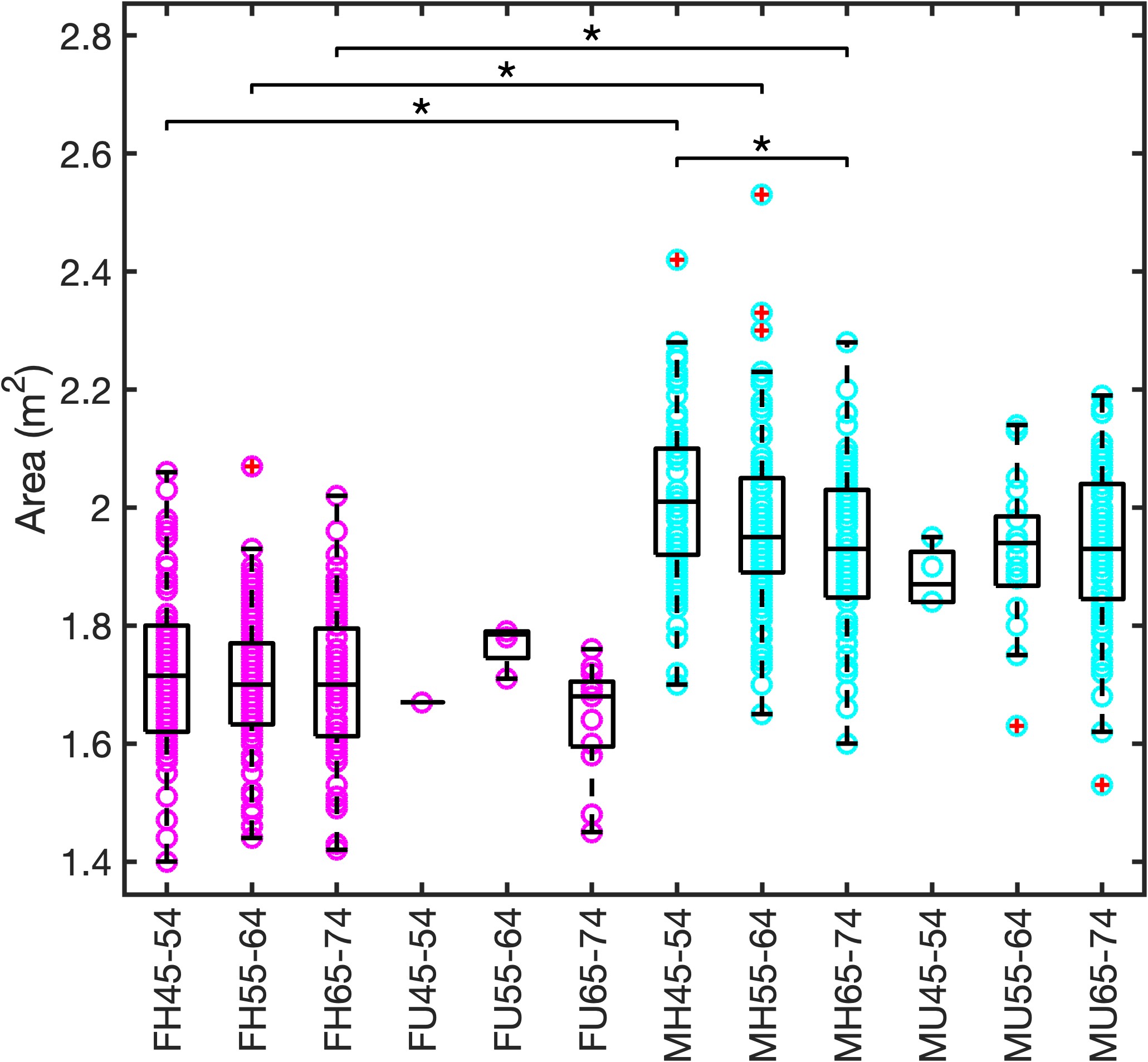


Figure 4: BSA as boxplots for all groups: M = Male; F = Female; H = Healthy; U = Unhealthy; XX-YY = age range. Two further planned comparisons were statistically significant that could not be represented on this plot: all healthy females vs. all healthy males, and all unhealthy females vs. all unhealthy males.


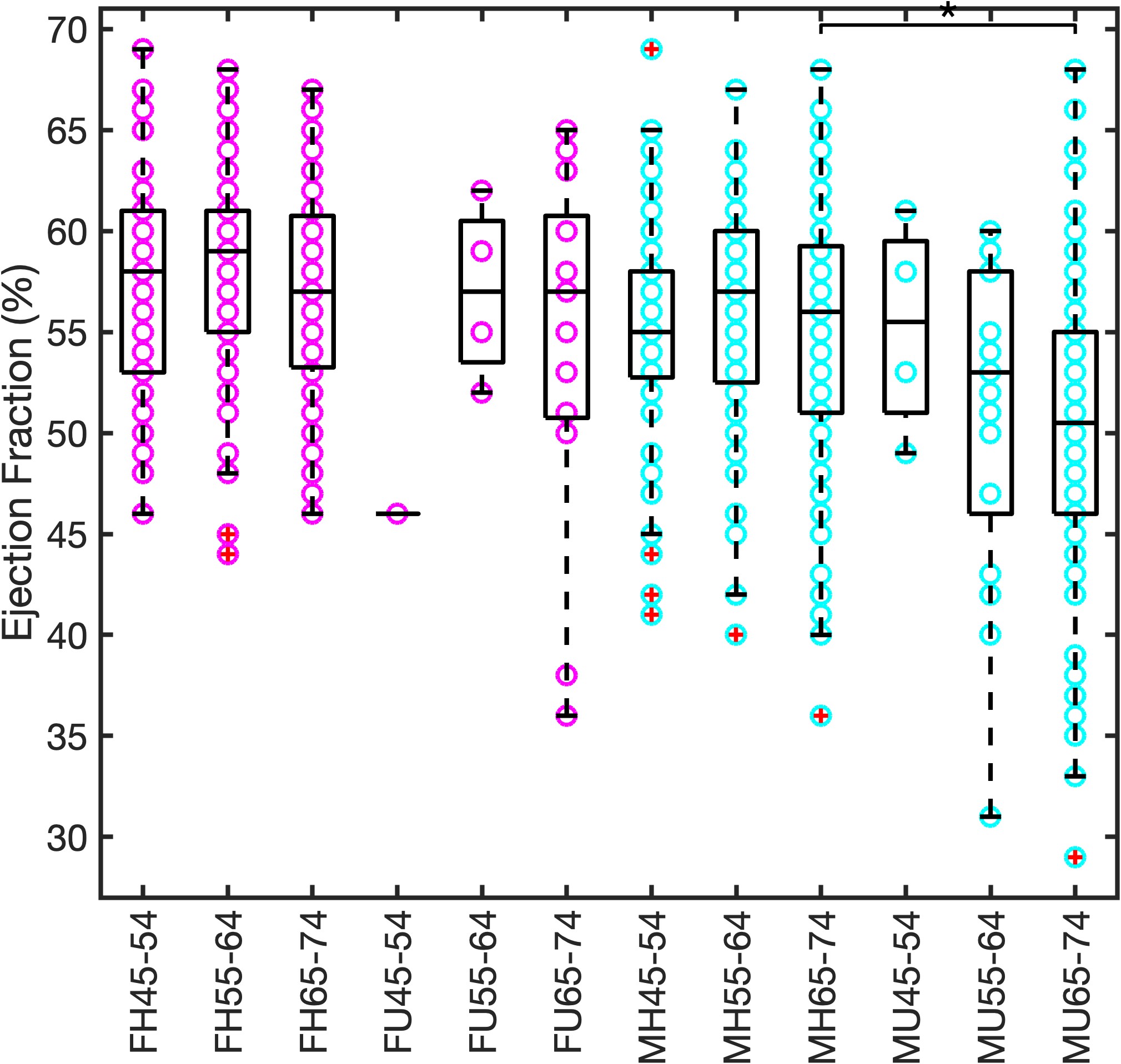


Figure 5: LVEF boxplots for all groups: M = Male; F = Female; H = Healthy; U = Unhealthy; XX-YY = age range. One further planned comparison was statistically significant that cannot be represented on this plot: all healthy females vs. all healthy males.
