# Supplementary material for: MRI‐based strain measurements reflect morphological changes following myocardial infarction: A study on the UK Biobank cohort
Source: J Anat. 2022 Dec 9;242(1):102–11. doi: 10.1111/joa.13787 (PMC9773168; doi:10.1111/joa.13787)
Supplement: Supplementary file 7 — Table S3 [file JOA-242-102-s004.pdf]

Online Supplementary Materials

Table 1: Results from Student's t-test for all males vs. all females, as well as all healthy vs. all unhealthy participants. All results were statistically significant.

Variable	All Males vs All Females p-values	All Healthy vs All Unhealthy p-values
ESV	0.001	0.001
EDV	0.001	0.001
LVEF	0.001	0.001
ESVi	0.001	0.001
BSA	0.001	0.005
ACS	0.002	0.006
MCS	0.003	0.001
BCS	0.001	0.001
GCS	0.001	0.001

Table 2: Further data for the Student's t-test for all male vs. all female volunteers. Where the Variance p-value is less than 0.05, the data shown assumes that the variances between the groups was not equal.

Variables	Variance p-value	Mean Difference	Std. Error Difference	95% Confidence Interval of the Difference	
				Lower	Upper
ESV	0	-22.5	1.3	-25.2	-19.9
EDV	0	-38.9	2.1	-43.0	-34.9
LVEF	0.002	3.3	0.5	2.4	4.2
ESVi	0	-7.3	0.7	-8.7	-6.0
BSA	0.06	-0.3	0.01	-0.3	-0.2
ACS	0.03	-2.1	0.7	-3.4	-0.8
MCS	0	-1.1	0.4	-1.8	-0.4
BCS	0.2	-2.6	0.4	-3.4	-1.8
GCS	0.001	-1.8	0.4	-2.6	-1.1

Table 3: Further data for the Student's t-test for all healthy vs. all unhealthy volunteers. Where the Variance p-value is less than 0.05, the data shown assumes that the variances between the groups was not equal.

Variables	Variance p-value	Mean Difference	Std. Error Difference	95% Confidence Interval of the Difference	
				Lower	Upper
ESV	0	-17.9	2.8	-23.4	-12.4
EDV	0.07	-18.3	3.3	-24.9	-11.8
LVEF	0	5.4	0.8	3.9	6.9
ESVi	0	-8.7	1.4	-11.4	-6.0
BSA	0.003	-0.05	0.02	-0.08	-0.01
ACS	0	-3.5	1.2	-5.9	-1.0
MCS	0	-2.1	0.6	-3.4	-0.9
BCS	0	-2.3	0.7	-3.7	-1.0
GCS	0	-2.8	0.7	-4.2	-1.4

Table 4: Results from the initial one-way ANOVAs. All p-values were less than the required 0.05.

ANOVA	p-value
ESV	0.001
EDV	0.001
LVEF	0.001
ESVi	0.001
BSA	0.001
ACS	0.019
MCS	0.001
BCS	0.001
GCS	0.001

Table 5: Baseline characteristics for healthy females of all ages.

Measure	n	Mean	Median	St. Dev.	Lower 95% CI	Upper 95% CI
BMI (kg/m^2)	281	24.53	24.38	2.78	24.2	24.86
HR (bpm)	260	62.09	61	9.22	60.95	63.23
Weight (kg)	281	65.51	64.7	8.34	64.51	66.51
Age (years)	281	60.17	61	7.58	59.27	61.07
ESV (mL)	260	52.92	52	11.6	51.49	54.35
EDV (mL)	260	124.2	124	21.2	121.5	126.8
LVEF (%)	260	57.42	58	4.91	56.81	58.03
ESVi (mL/m^2)	260	31.01	30.09	6.4	30.22	31.8
BSA (m^2)	260	1.71	1.7	0.12	1.7	1.72
ACS (%)	281	-42.43	-42.17	7.68	-43.35	-41.51
MCS (%)	281	-29.68	-29.74	3.9	-30.15	-29.21
BCS (%)	281	-32.8	-32.42	5.01	-33.4	-32.2
GCS (%)	281	-34.29	-34.4	4.09	-34.78	-33.8

Table 6: Baseline characteristics for unhealthy females of all ages.

Measure	n	Mean	Median	St. Dev.	Lower 95% CI	Upper 95% CI
BMI (kg/m^2)	20	24.85	24.73	2.84	23.58	26.12
HR (bpm)	18	61.56	58	10.9	56.43	66.69
Weight (kg)	20	63.6	64.25	7.28	60.34	66.86
Age (years)	20	66.85	68.5	5.03	64.6	69.1
ESV (mL)	18	58.11	57	17.7	49.75	66.47
EDV (mL)	18	126.6	129	24	115.3	137.9
LVEF (%)	18	54.56	56	8.18	50.7	58.42
ESVi (mL/m^2)	18	34.66	34.13	10.5	29.73	39.59
BSA (m^2)	18	1.67	1.69	0.1	1.62	1.72
ACS (%)	20	-39.97	-42.74	13.9	-46.18	-33.76
MCS (%)	20	-27.98	-29.15	5.44	-30.41	-25.55
BCS (%)	20	-30.94	-30.64	7.16	-34.14	-27.74
GCS (%)	20	-32.13	-33.26	7.65	-35.55	-28.71

Table 7: Baseline characteristics for healthy males of all ages.

Measure	n	Mean	Median	St. Dev.	Lower 95% CI	Upper 95% CI
BMI (kg/m^2)	293	25.65	25.9	2.42	25.37	25.93
HR (bpm)	274	60.32	60	8.86	59.25	61.39
Weight (kg)	293	80.45	80.4	9.5	79.34	81.56
Age (years)	293	60.35	60	7.87	59.43	61.27
ESV (mL)	274	72.4	70	16.8	70.37	74.43
EDV (mL)	274	161.4	159	29	157.9	165
LVEF (%)	274	55.3	56	5.59	54.62	55.98
ESVi (mL/m^2)	274	36.56	35.98	7.85	35.61	37.51
BSA (m^2)	274	1.98	1.97	0.14	1.96	2
ACS (%)	293	-41.08	-40.86	7.9	-42	-40.16
MCS (%)	293	-29.08	-28.67	4.35	-29.59	-28.57
BCS (%)	293	-30.53	-30.73	3.89	-30.98	-30.08
GCS (%)	293	-32.99	-32.99	4.44	-33.51	-32.47

Table 8: Baseline characteristics for unhealthy males of all ages.

Measure	n	Mean	Median	St. Dev.	Lower 95% CI	Upper 95% CI
BMI (kg/m^2)	111	25.68	25.78	2.28	25.25	26.11
HR (bpm)	101	56.02	54	9.04	54.22	57.82
Weight (kg)	111	78.19	78.3	8.65	76.55	79.83
Age (years)	111	66.8	68	5.36	65.78	67.82
ESV (mL)	101	84.88	78	28.8	79.14	90.62
EDV (mL)	101	167.9	161	37	160.5	175.2
LVEF (%)	101	50.25	51	7.68	48.72	51.78
ESVi (mL/m^2)	101	44	40.51	14.8	41.06	46.94
BSA (m^2)	101	1.93	1.93	0.13	1.9	1.96
ACS (%)	111	-37.97	-41.54	13.8	-40.6	-35.34
MCS (%)	111	-27.1	-27.81	7.4	-28.5	-25.7
BCS (%)	111	-29	-28.28	7.45	-30.41	-27.59
GCS (%)	111	-30.61	-32.14	7.75	-32.08	-29.14

Table 9: Mean time in years since initial cardiac event. * There was only one participant in this group.

Unhealthy Group	Mean time (years)
Females 45-54	14.7*
Females 55-64	2.8
Females 65-74	4.3
Males 45-54	4.6
Males 55-64	5.9
Males 65-74	7.7

Table 12: Age and BSA correlations with other variables of interest for all healthy females. Statistically significant p-values are highlighted in green. The 95% CIs are taken from the bootstrapping analysis.

Variable	Age Correlations				
	n	Pearson	p-value	95% CIs	
				Lower	Upper
EDV	260	-0.193	0.002	-0.303	-0.069
ESV	260	-0.120	0.054	-0.243	-0.003
LVEF	260	-0.059	0.344	-0.189	0.068
ESVi	260	-0.120	0.054	-0.242	0.008
ACS	281	0.046	0.442	-0.089	0.175
MCS	281	-0.163	0.006	-0.272	-0.029
BCS	281	-0.006	0.918	-0.114	0.134
GCS	281	-0.041	0.490	-0.156	0.090
Variable	BSA Correlations				
EDV	260	0.443	0.000	0.341	0.542
ESV	260	0.318	0.000	0.202	0.425
LVEF	260	0.065	0.300	-0.070	0.190
ESVi	260	-0.017	0.786	-0.137	0.109
ACS	260	-0.046	0.459	-0.165	0.074
MCS	260	-0.063	0.308	-0.208	0.071
BCS	260	0.054	0.384	-0.065	0.153
GCS	260	-0.007	0.917	-0.136	0.117

Table 10: P-values for all strain and strain rate metrics. Results highlighted in green with bold text and *, indicates results which are statistically significant to $p \leq 0.003$.

Planned Comparison	ACS	MCS	BCS	GCS
Female Healthy 45-54 vs. Female Healthy 55-64	0.556	0.041	0.462	0.439
Female Healthy 45-54 vs. Female Healthy 65-74	0.816	**0.002***	0.785	0.226
Female Healthy 55-64 vs. Female Healthy 65-74	0.412	0.272	0.245	0.669
Female Healthy 45-54 vs. Male Healthy 45-54	0.321	0.569	**0.001***	0.066
Female Healthy 55-64 vs. Male Healthy 55-64	0.078	0.376	0.004	0.051
Female Healthy 65-74 vs. Male Healthy 65-74	0.449	0.153	**0.001***	0.015
Male Healthy 45-54 vs. Male Healthy 55-64	0.839	0.098	0.505	0.682
Male Healthy 45-54 vs. Male Healthy 65-74	0.969	0.069	0.709	0.824
Male Healthy 55-64 vs. Male Healthy 65-74	0.860	0.763	0.762	0.865
Male Healthy 65-74 vs. Male Unhealthy 65-74	0.033	**0.001***	0.118	0.01
Female Healthy 65-74 vs. Female Unhealthy 65-74	0.725	0.145	0.266	0.311
Male Healthy 55-64 vs. Male Unhealthy 55-64	0.790	0.563	0.147	0.303
Male Unhealthy 55-64 vs. Male Unhealthy 65-74	0.436	0.369	0.857	0.569
All Males Healthy vs. All Females Healthy	0.060	0.061	**0.001***	**0.001***
All Females Healthy vs. All Females Unhealthy	0.437	0.248	0.305	0.283
All Males Healthy vs. All Males Unhealthy	0.299	0.513	0.207	0.228
All Females Unhealthy vs. All Males Unhealthy	0.948	0.719	0.288	0.877

Table 11: P-values for all strain and strain rate metrics. Results highlighted in green with bold text and *, indicates results which are statistically significant to $p \leq 0.003$.

Planned Comparison	LVEF	EDV	ESV	ESVi	BSA
Female Healthy 45-54 vs. Female Healthy 55-64	0.252	**0.003***	0.004	0.010	0.196
Female Healthy 45-54 vs. Female Healthy 65-74	0.872	**0.002***	0.024	0.040	0.315
Female Healthy 55-64 vs. Female Healthy 65-74	0.209	0.693	0.711	0.781	0.843
Female Healthy 45-54 vs. Male Healthy 45-54	0.020	**0.001***	**0.001***	**0.001***	**0.001***
Female Healthy 55-64 vs. Male Healthy 55-64	0.005	**0.001***	**0.001***	**0.001***	**0.001***
Female Healthy 65-74 vs. Male Healthy 65-74	0.017	**0.001***	**0.001***	**0.001***	**0.001***
Male Healthy 45-54 vs. Male Healthy 55-64	0.755	0.005	0.150	0.053	0.048
Male Healthy 45-54 vs. Male Healthy 65-74	0.516	**1.00E-03***	**0.003***	0.039	**0.001***
Male Healthy 55-64 vs. Male Healthy 65-74	0.357	0.070	0.392	0.701	0.071
Male Healthy 65-74 vs. Male Unhealthy 65-74	**0.001***	0.018	**0.001***	**0.001***	0.886
Female Healthy 65-74 vs. Female Unhealthy 65-74	0.351	0.962	0.499	0.346	0.074
Male Healthy 55-64 vs. Male Unhealthy 55-64	0.031	0.008	0.013	0.008	0.111
Male Unhealthy 55-64 vs. Male Unhealthy 65-74	0.485	0.094	0.734	0.632	0.701
All Males Healthy vs. All Females Healthy	**0.001***	**0.001***	**0.001***	**0.001***	**0.001***
All Females Healthy vs. All Females Unhealthy	0.202	0.248	0.209	0.206	0.574
All Males Healthy vs. All Males Unhealthy	0.044	0.151	0.074	0.052	0.004
All Females Unhealthy vs. All Males Unhealthy	0.263	**0.003***	0.012	0.090	**0.001***

Table 13: Age and BSA correlations with other variables of interest for all unhealthy females. Statistically significant p-values are highlighted in green. The 95% CIs are taken from the bootstrapping analysis.

Variable	Age Correlations				
	n	Pearson	p-value	95% CIs	
				Lower	Upper
EDV	18	-0.49	0.039	-0.790	0.012
ESV	18	-0.319	0.196	-0.243	-0.003
LVEF	18	0.005	0.984	-0.517	0.672
ESVi	18	-0.269	0.281	-0.781	0.351
ACS	20	-0.187	0.431	-0.682	0.390
MCS	20	-0.165	0.487	-0.613	0.365
BCS	20	0.093	0.697	-0.149	0.506
GCS	20	-0.123	0.606	-0.567	0.370
Variable	BSA Correlations				
EDV	18	0.428	0.076	-0.148	0.721
ESV	18	0.307	0.216	0.202	0.425
LVEF	18	-0.155	0.538	-0.594	0.526
ESVi	18	0.157	0.534	-0.525	0.642
ACS	18	0.113	0.656	-0.358	0.470
MCS	18	0.131	0.605	-0.403	0.552
BCS	18	0.053	0.834	-0.477	0.471
GCS	18	0.131	0.605	-0.390	0.506

Table 14: Age and BSA correlations with other variables of interest for all healthy males. Statistically significant p-values are highlighted in green. The 95% CIs are taken from the bootstrapping analysis.

Variable	Age Correlations				
	n	Pearson	p-value	95% CIs	
				Lower	Upper
EDV	274	-0.280	0.000	-0.399	-0.152
ESV	274	-0.192	0.001	-0.316	-0.063
LVEF	274	-0.047	0.439	-0.171	0.082
ESVi	274	-0.133	0.028	-0.249	-0.003
ACS	293	-0.026	0.657	-0.186	0.062
MCS	293	-0.160	0.006	-0.308	-0.072
BCS	293	-0.033	0.569	-0.139	0.088
GCS	293	-0.048	0.411	-0.201	0.050
Variable	BSA Correlations				
EDV	274	0.482	0.000	0.390	0.567
ESV	274	0.395	0.000	0.293	0.484
LVEF	274	-0.049	0.420	-0.168	0.070
ESVi	274	0.100	0.097	-0.012	0.207
ACS	274	0.075	0.217	-0.029	0.183
MCS	274	0.170	0.005	0.049	0.283
BCS	274	0.092	0.130	-0.044	0.210
GCS	274	0.108	0.074	-0.008	0.226

Table 15: Age and BSA correlations with other variables of interest for all unhealthy males. Statistically significant p-values are highlighted in green. The 95% CIs are taken from the bootstrapping analysis.

Variable	Age Correlations				
	n	Pearson	p-value	95% CIs	
				Lower	Upper
EDV	101	-0.114	0.256	-0.315	0.065
ESV	101	0.049	0.626	-0.159	0.200
LVEF	101	-0.204	0.041	-0.374	-0.026
ESVi	101	0.048	0.632	-0.151	0.215
ACS	111	0.089	0.351	-0.049	0.339
MCS	111	0.148	0.120	-0.064	0.342
BCS	111	0.020	0.835	-0.131	0.192
GCS	111	0.101	0.293	-0.056	0.320
Variable	BSA Correlations				
EDV	101	0.312	0.001	0.138	0.471
ESV	101	0.165	0.100	-0.001	0.339
LVEF	101	0.074	0.465	-0.107	0.266
ESVi	101	-0.039	0.697	-0.197	0.150
ACS	101	-0.169	0.092	-0.342	0.013
MCS	101	-0.112	0.263	-0.315	0.098
BCS	101	-0.128	0.201	-0.329	0.091
GCS	101	-0.160	0.109	-0.343	0.024

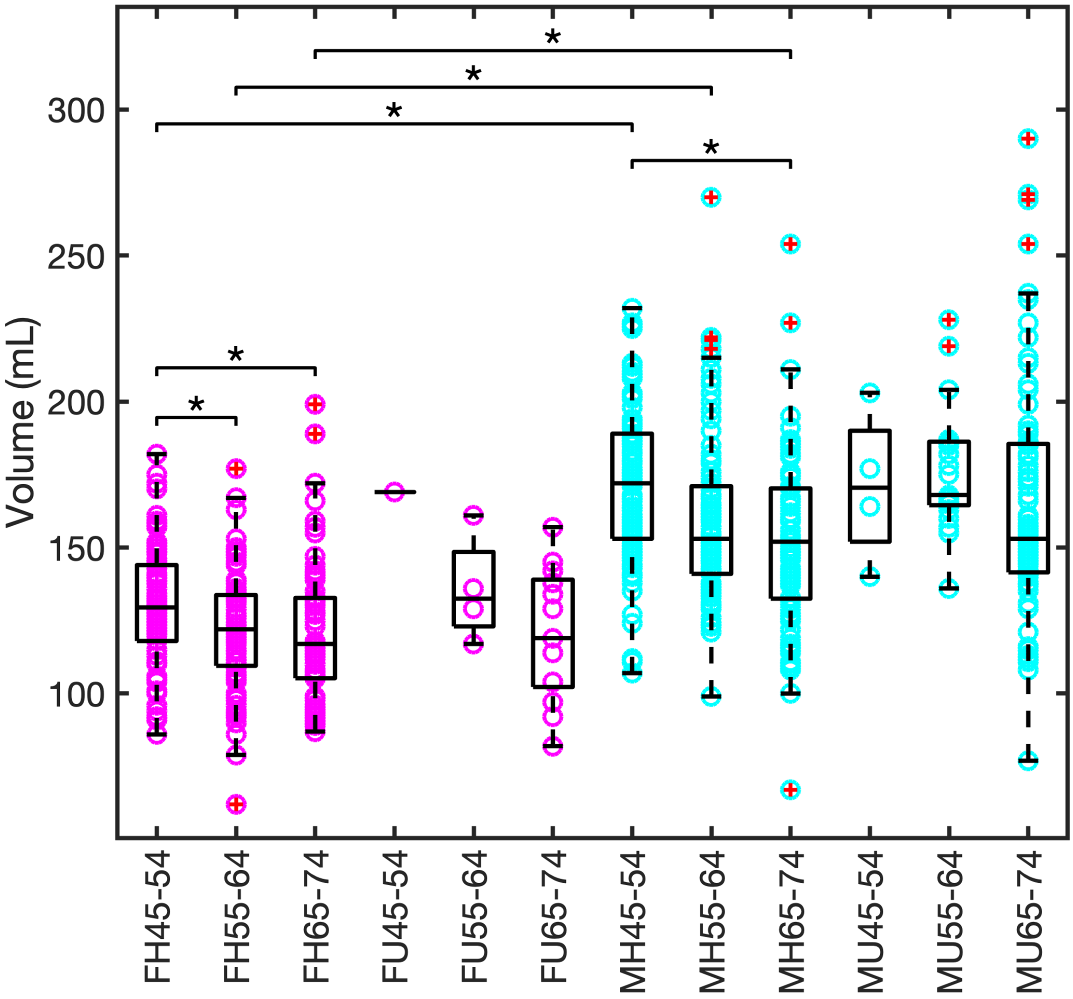

Figure 1: EDV boxplots for all groups: M = Male; F = Female; H = Healthy; U = Unhealthy; XX-YY = age range. Two further planned comparisons were statistically significant that could not be represented: all healthy females vs. all healthy males, and all unhealthy females vs. all unhealthy males.

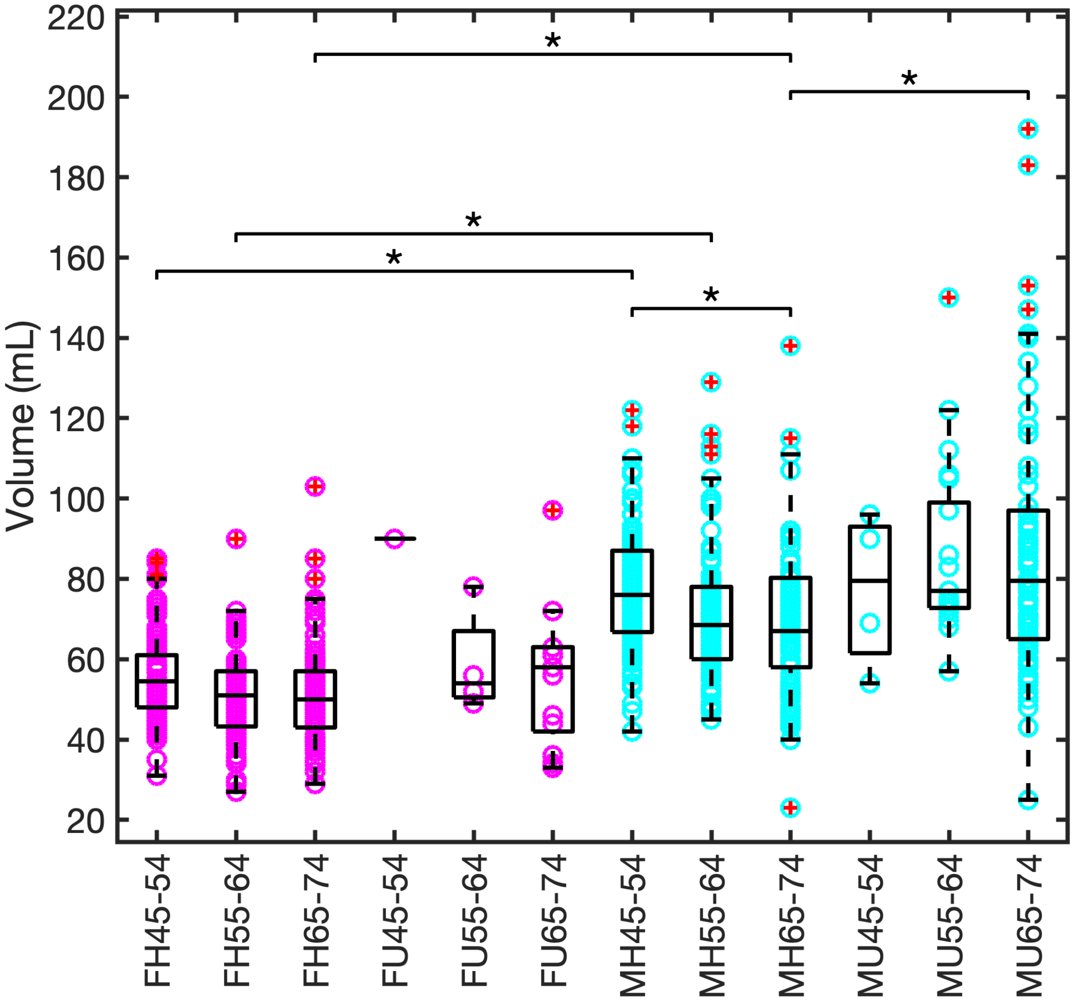

Figure 2: ESV boxplots for all groups: M = Male; F = Female; H = Healthy; U = Unhealthy; XX-YY = age range. One further planned comparison was statistically significant that could not be represented on this plot: all healthy females vs. all healthy males.

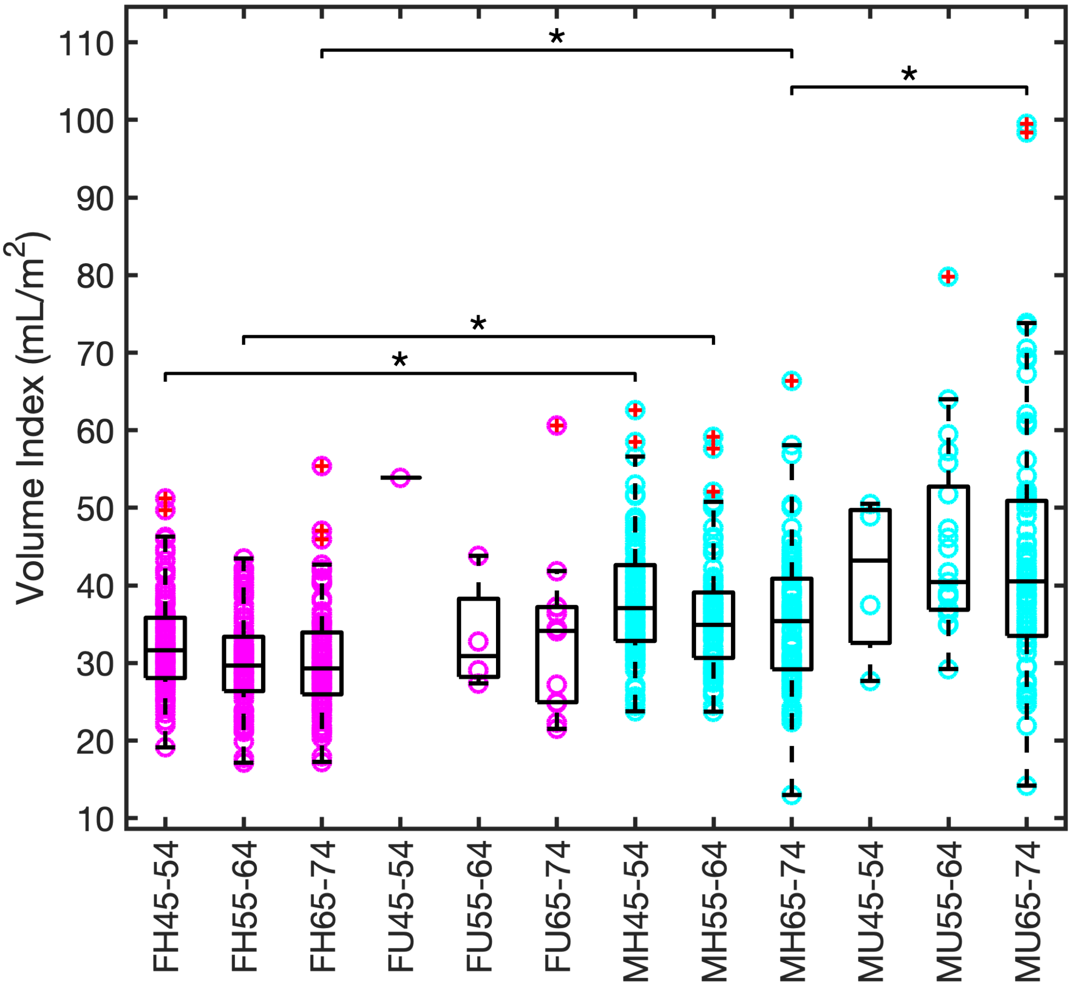

Figure 3: ESVi as boxplots for all groups: M = Male; F = Female; H = Healthy; U = Unhealthy; XX-YY = age range. One further planned comparison was statistically significant that could not be represented on this plot: all healthy females vs. all healthy males.

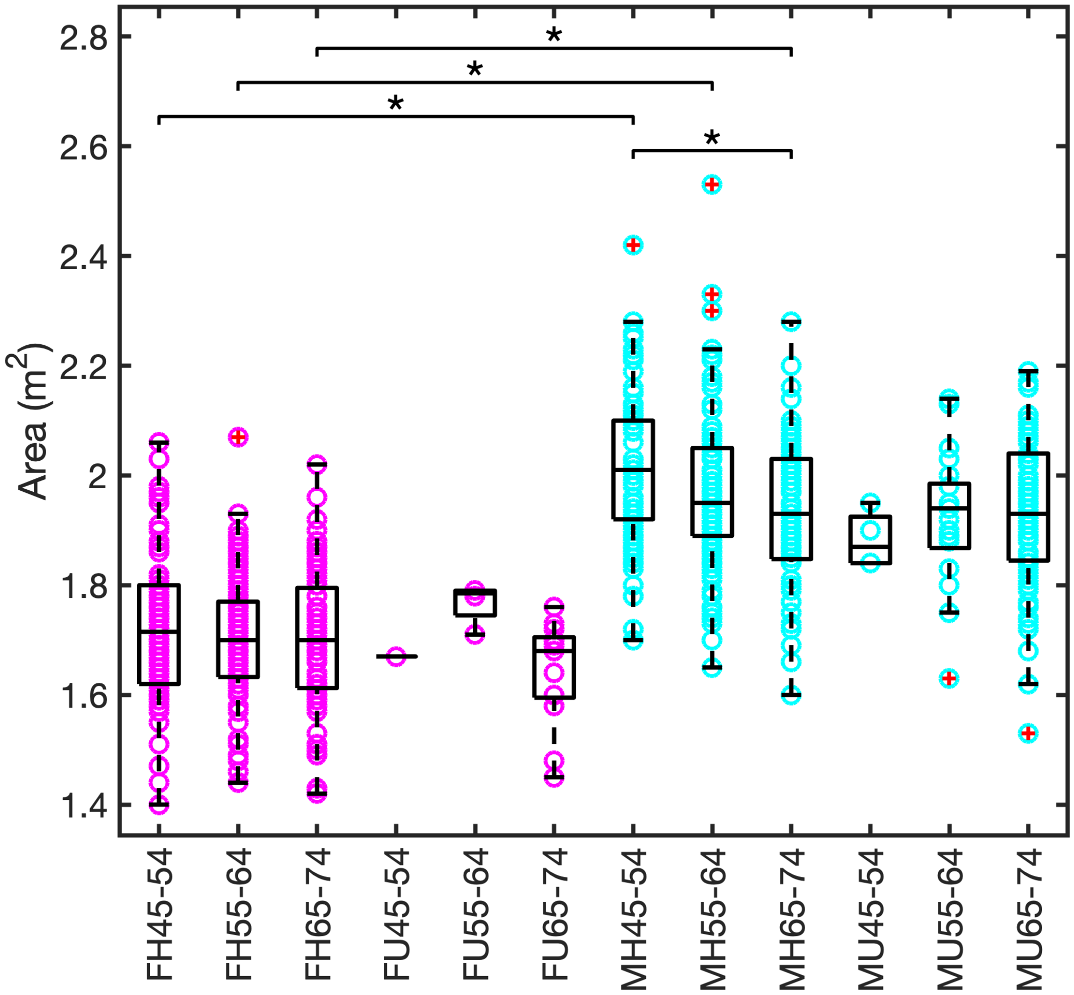

Figure 4: BSA as boxplots for all groups: M = Male; F = Female; H = Healthy; U = Unhealthy; XX-YY = age range. Two further planned comparisons were statistically significant that could not be represented on this plot: all healthy females vs. all healthy males, and all unhealthy females vs. all unhealthy males.

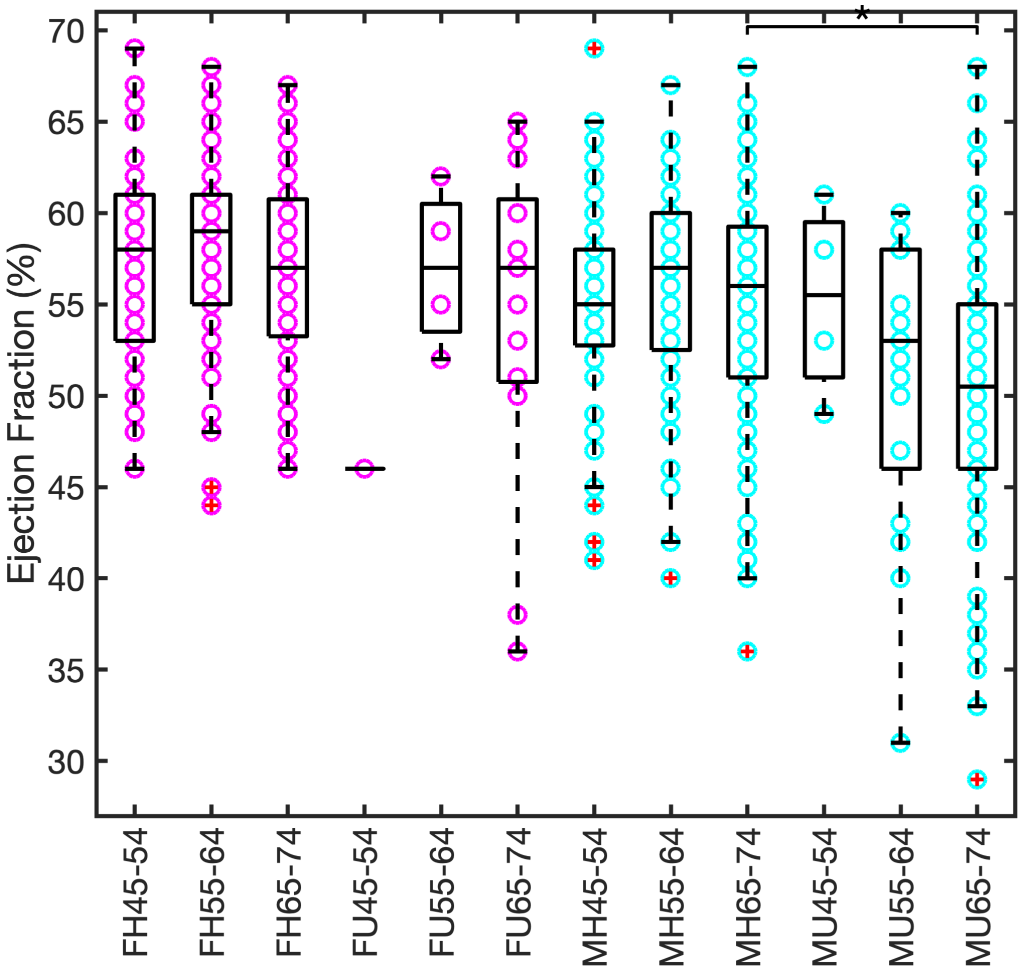

Figure 5: LVEF boxplots for all groups: M = Male; F = Female; H = Healthy; U = Unhealthy; XX-YY = age range. One further planned comparison was statistically significant that cannot be represented on this plot: all healthy females vs. all healthy males.
